# Synthesis, In Silico Studies, and Evaluation of Syn and Anti Isomers of *N*-Substituted Indole-3-carbaldehyde Oxime Derivatives as Urease Inhibitors against *Helicobacter pylori*

**DOI:** 10.3390/molecules26216658

**Published:** 2021-11-03

**Authors:** Ishani P. Kalatuwawege, Medha J. Gunaratna, Dinusha N. Udukala

**Affiliations:** 1 College of Chemical Sciences, Institute of Chemistry Ceylon, Rajagiriya 10100, Sri Lanka; ishanisjc@gmail.com (I.P.K.); dinusha@ichemc.edu.lk (D.N.U.); 2 Department of Chemistry, University of Kelaniya, Kelaniya 10300, Sri Lanka

**Keywords:** *Helicobacter pylori*, urease inhibitors, indole-3-carbaldehyde oximes, isomerization, molecular docking

## Abstract

Gastrointestinal tract infection caused by *Helicobacter pylori* is a common virulent disease found worldwide, and the infection rate is much higher in developing countries than in developed ones. In the pathogenesis of *H. pylori* in the gastrointestinal tract, the secretion of the urease enzyme plays a major role. Therefore, inhibition of urease is a better approach against *H. pylori* infection. In the present study, a series of syn and anti isomers of *N*-substituted indole-3-carbaldehyde oxime derivatives was synthesized via Schiff base reaction of appropriate carbaldehyde derivatives with hydroxylamine hydrochloride. The in vitro urease inhibitory activities of those derivatives were evaluated against that of *Macrotyloma uniflorum* urease using the modified Berthelot reaction. Out of the tested compounds, compound **8** (IC_50_ = 0.0516 ± 0.0035 mM) and compound **9** (IC_50_ = 0.0345 ± 0.0008 mM) were identified as the derivatives with potent urease inhibitory activity with compared to thiourea (IC_50_ = 0.2387 ± 0.0048 mM). Additionally, in silico studies for all oxime compounds were performed to investigate the binding interactions with the active site of the urease enzyme compared to thiourea. Furthermore, the drug-likeness of the synthesized oxime compounds was also predicted.

## 1. Introduction

Gastrointestinal tract infections are one of the most prevalent and problematic clinical disorders worldwide. Usually, they are associated with acute and chronic gastritis, gastroparesis, dyspepsia, gastropathy, peptic ulcers, and different kinds of cancers that have an impact on digestion and the overall health of humans [1,2,3]. However, it is known that most of the stomach related infections are caused by a bacterium known as *Helicobacter pylori*, which is mostly notable for its high level of urease activity [4,5]. Urease (urea amidohydrolase; EC 3.5.1.5) is a non-redox nickel-containing enzyme with a high molecular weight which is found in many organisms such as plants, algae, fungi, bacteria, and even in some invertebrates, enabling them to utilize urea as a nitrogen source [2,6,7]. *H. pylori’s *urease has a major role in the virulence of the bacteria as an etiological factor of diseases affecting the upper intestinal tract [2,7,8]. During infection, *H. pylori* must pass through gastric acid to reach the protective mucin layer [8]. *H. pylori* urease catalyzes the hydrolysis of urea to ammonia and carbon dioxide [2,9]. Protonation of the produced ammonia results in pH increment, and thereby, the protective gel layer (mucin) of the gastric epithelium undergoes a reversible sol-gel transition [10]. This process reduces the viscosity of the gel and enables *H. pylori* to swim freely through the gel and infect the gastric epithelium [10]. Additionally, it has been suggested that chemotactic motility is important for the colonization of *H. pylori* [11]. Therefore, inhibition of the urease enzyme of *H. pylori* can arrest its growth, colonization, and survival in the gastric acidic environments [2,12].

The inhibitory activity of the urease enzyme has been studied extensively because of its potential uses in the treatment of gastrointestinal tract infections [2]. Hydroxamic acid [13], phosphoramidate [2,14], thiourea [15], benzimidazole [11], hydroxyurea [16], and their derivatives are reported as potent urease inhibitors. Among them, acetohydroxamic acid, which belongs to the hydroxamic acid group, exhibits high inhibitory activity by obstructing chemotactic moments of *H. pylori* and by exerting bacteriostatic and bactericidal effects on *H. pylori* [2,11,13]. However, it has been reported to cause side effects such as neurological disorders and teratogenicity [11]. Few alternative drugs, including omeprazole, rabeprazole, and lansoprazole, have been developed to eradicate the bacteria when combined with antibiotics such as amoxicillin, clarithromycin, and metronidazole [11]. Apart from urease inhibitors, these antibiotic drugs have been used to treat *H. pylori* infections. However, regardless of the efficacy, these drugs should be given in higher doses as they tend to degrade by gastric acid [17]. As a result of that, several side effects such as abdominal pain, nausea, vomiting, and diarrhea can occur [17]. It can be seen that the effectiveness of most of the existing anti urease drugs is limited by their toxicity and the possible development of resistance. Therefore, it is essential to develop novel, selective, and effective treatment strategies using urease inhibitors that satisfy the low toxicity requirement for human health. 

Oximes are a class of nitrogen-containing compounds that can be prepared by a Schiff base formation reaction. According to the literature [18], there are three types of tautomeric forms: the oxime, the nitrone, and the nitroso compound. Among them, oxime exists as the most stable form [18].

In this study, five *N*-substituted indole-3-carbaldehyde oxime derivatives were synthesized as syn (Z) and anti (E) isomers due to their structural similarities to acetohydroxamic acid, which is a potent urease inhibitor. Their urease inhibitory activities were determined using the modified Berthelot reaction. Molecular docking studies were carried out to identify the binding interactions of these compounds with the active site of the urease enzyme of *Helicobacter pylori*. Additionally, the drug-likeness of the oxime derivatives was predicted using SwissADME.

## 2. Results and Discussion

### 2.1. Chemistry

Five compounds of *N*-substituted indole-3-carboxaldehyde oxime derivatives (including syn and anti isomers) were synthesized in the solution-phase. To study the isomerization process and identify the syn and anti configurations of the resulting oxime compounds, the synthesis of oximes was carried out under different reaction conditions. Since excess NH_2_OH.HCl has been used in this reaction in the reported literature [19,20,21,22,23], it can be hypothesized that increase in the amount of NH_2_OH.HCl and NaOH would lead to improved yield. Therefore, in this study, up to five equivalents of an oxygen base (NaOH) and five equivalents of NH_2_OH.HCl were used in the oximation reactions of *N*-substituted indole-3-carboxaldehyde oximes. 

**Scheme 1:**
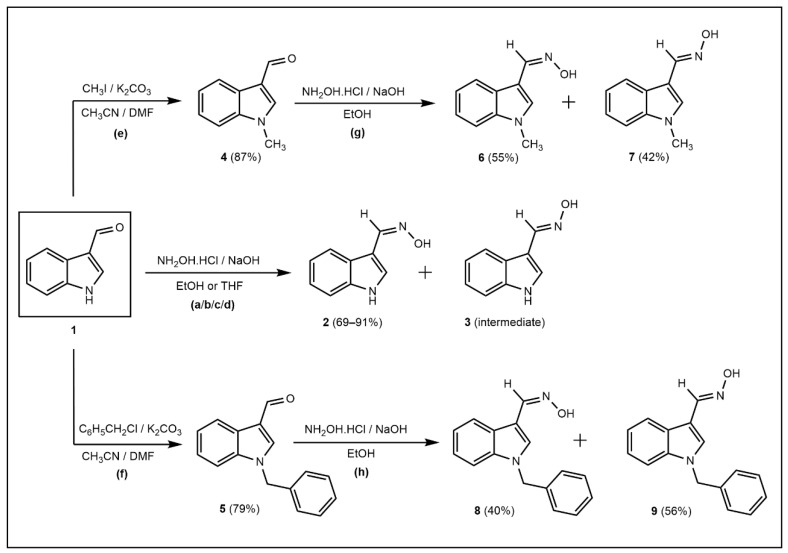
Synthesis of indole-3-carbaldehyde oxime derivatives and their intermediates (Compound **2**–**9**). Reagents and conditions: (**a**) NH_2_OH.HCl (5 eq), aq. NaOH (2 eq), 95% EtOH, 0–27 °C, 2 h; (**b**) NH_2_OH.HCl (5 eq), aq. NaOH (5 eq), 95% EtOH, 0–27 °C, 2 h; (**c**) NH_2_OH.HCl (5 eq), aq. NaOH (2 eq), THF, 0–27 °C, 4 h and 15 min; (**d**) NH_2_OH.HCl (5 eq), aq. NaOH (5 eq), THF, 0–27 °C, 4 h and 15 min; (**e**) CH_3_I, anh. K_2_CO_3,_ CH_3_CN, DMF, 82–84 °C, 16 h; (**f**) C_6_H_5_CH_2_Cl, anh. K_2_CO_3_, CH_3_CN, DMF, 82–84 °C, 12 h; (**g**) NH_2_OH.HCl (5 eq), NaOH (5 eq), 95% EtOH, H_2_O, 0–27 °C, 4 h; (**h**) NH_2_OH.HCl (5 eq), NaOH (5 eq), 95% EtOH, H_2_O, 0–27 °C, 2 h.

Initially, (*Z*)-*N*-hydroxy-1-(1*H*-indole-3-yl)methanimine (**2**) was synthesized via a condensation reaction using 1*H*-indole-3-carbaldehyde (**1**) dissolved in an appropriate solvent (95% ethanol or THF) and hydroxylamine hydrochloride (NH_2_OH.HCl) dissolved in a solution of NaOH at 0–27 °C for 2–4 h [19,21]. This reaction was conducted under both acidic and neutral conditions separately by changing the ratios of NH_2_OH.HCl and NaOH (Scheme 1).

The effect of the solvent on the oximation reaction was investigated using polar protic ethanol and polar aprotic THF. The reaction was completed in a lesser time with the highest yield when it was carried out in ethanol compared to THF. The capability of ethanol as a polar protic solvent in forming hydrogen bonding with the reactants would enhance the rate of the reaction and improve the yield. However, no significant effect was observed on the oximation reaction when the pH of the medium was changed. 

Even though non-substituted indole-3-carbaldehyde oximes resulted in only the syn isomer in literature [19,22,24,25], we observed both compound **2** (syn oxime) and compound **3** (intermediate, anti oxime) upon completion of the above-mentioned oximation reactions. Since the stability of the anti isomers of indole-3-carbaldehyde oxime derivatives is considerably low in an acidic medium, isomerization of the anti product into the syn product of those oximes are highly favorable [19,26,27]. Therefore, reactions **a** (89.07%) and **c** (68.93%) were carried out under acidic conditions and **b** (91.29%) and **d** (74.21%) under neutral conditions by altering the amount of NaOH to investigate the effect of the pH of the reaction medium on the isomerization process. 

**Figure 1:**
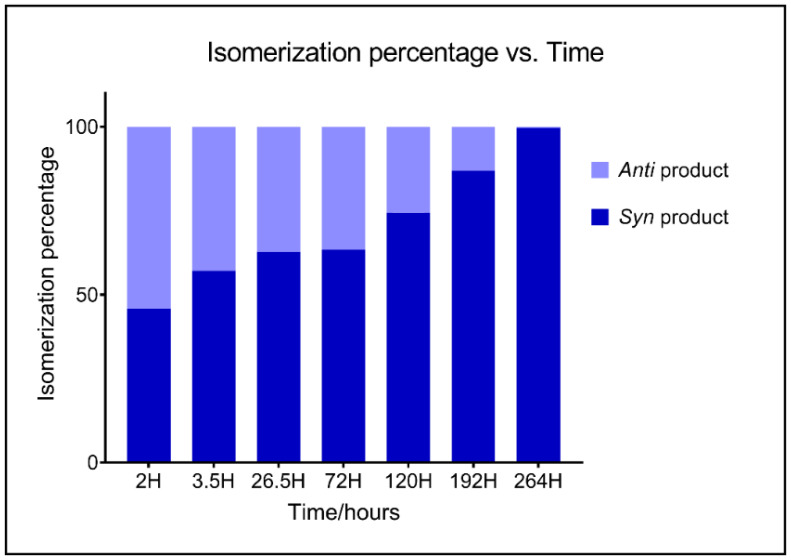
Percentage isomerization vs. time of (*Z*/*E*)-*N*-hydroxy-1-(1*H*-indole-3-yl)methanimine (compounds **2** and **3**) under acidic medium.

According to the time-resolved TLC analysis (Figure S1), it was observed that the spot of compound **3** gradually disappeared while intensifying the spot of compound **2** over time, regardless of the pH of the reaction medium and the solvent. In reaction **a**, the time consumed for the completion of the isomerization was less than that for reaction **b**. Similarly, reaction **c** consumed less time than reaction **d**. Therefore, it can be concluded that the isomerization process is accelerated under acidic conditions. The progress of the isomerization process was monitored by calculating the percentages of both syn and anti oximes (compounds **2** and **3**) using ImageJ software (U.S. National Institutes of Health: Bethesda, MD, USA) [28]. As per the literature [29], it has been proven that this analysis method is compatible with the other densitometric analysis methods. The calculated percentages of both syn and anti isomers of *N*-hydroxy-1-(1*H*-indole-3-yl)methanimine using the time-resolved TLC analysis are given in Figure 1.

The reaction conditions for *N*-alkylation of indole-3-carbaldehyde, in order to synthesize compounds **4** and **5**, were adapted from reported literature with some modifications (Scheme 1) [30,31]. *N*-alkylation of 1*H*-indole-3-carbaldehyde (**1**) using the corresponding alkyl halides in a mixture of acetonitrile and *N*,*N*-Dimethylformamide (DMF) under reflux for 12–16 h yielded 1-methyl-1*H*-indole-3-carbaldehyde (**4**) (reaction **e**) in 87.28% and 1-benzyl-1*H*-indole-3-carbaldehyde (**5**) (reaction **f**) in 78.81%. Since our main focus was to investigate the effect of both syn and anti oxime isomers against urease enzyme activity, we synthesized both isomers of *N*-substituted indole-3-carbaldehyde oxime derivatives using a neutral medium. Therefore, compounds **4** and **5** were used as the precursors for the synthesis of *N*-hydoxy-1-(1-methyl-1*H*-indole-3-yl)methylamine (compounds **6** and **7**) and *N*-hydoxy-1-(1-benzyl-1*H*-indole-3-yl)methylamine (compounds **8** and **9**), respectively, as mixtures of syn and anti isomers (Scheme 1) using similar conditions of reaction **b**. After the chromatographic separation, compound **6** was obtained in 54.77% yield, and compound **7** was obtained in 42.04% yield, whereas compound **8** was obtained in 40.12% yield, and compound **9** was obtained in 56.20% yield. 

All the synthesized oxime compounds were characterized using FT-IR (Figures S2–S7), ^1^H-NMR (Figures S8–S13), and ^13^C-NMR (Figures S14–S16) spectra. In the case of anti isomers, vibrations of the O-H bond were observed at a higher region than the O-H bond vibrations of syn isomers. The wavenumbers of these bonds in FT-IR were in accordance with the values reported in the literature [19]. According to ^1^H-NMR data, it was confirmed that the less polar compound **3** corresponds to the anti isomer and the more polar compound **2** as the syn isomer. The same trend was observed for compounds **6**, **7**, **8**, and **9**.

Considerable changes of the chemical shifts were observed for H-2, H-4, H-8, and OH protons of the syn and anti isomers (Table S1). When comparing the chemical shift values of the H-2, H-4, H-8, and OH protons of all syn compounds **2**, **6**, and **8**, it can be clearly seen that the compound **8**, which consists of a benzyl group, has the higher chemical shift values due to the anisotropic effect of the phenyl group compared to the methyl group. This phenomenon is well applied for the anti compounds **3**, **7**, and **9** as well.

### 2.2. Urease Inhibitory Assay 

All synthesized oxime compounds (**2**, **6**, **7**, **8**, and **9**) were evaluated for the in vitro urease inhibitory activity using a colorimetric method. This method involves the formation of indophenol blue in a basic medium where the modified Berthelot reaction is used [32,33]. The urease enzyme required for the assay was extracted from *Macrotyloma uniflorum* (Horse gram) with the activity of 0.1013 U, and thiourea was used as the standard inhibitor [34]. The amount of ammonia produced within a given period of time was quantified using a standard calibration method [35,36]. 

Urease inhibitory activities of the compounds **2**, **6**, **7**, **8**, and **9** are given in Table S2. They showed inhibition of the urease enzyme at the tested concentration range from 5.00 μg/mL to 35.00 μg/mL. The percentage inhibitions of all five tested compounds at 5 μg/mL and 10 μg/mL were higher than that of thiourea. Among these compounds, compound **9** showed more than 50% inhibition of urease at 10 μg/mL concentration, making it the most potent urease inhibitor. 

The IC_50_ values of compounds **2**, **6**, **7**, **8**, and **9** are given in Table 1. They possessed IC_50_ within the range of 0.0345 ± 0.0008–0.1530 ± 0.0016 mM, compared to thiourea (0.2387 ± 0.0048 mM). Compounds **8** and **9** are proved to be the most potent urease inhibitors with the IC_50_ values of 0.0516 ± 0.0035 mM and 0.0345 ± 0.0008 mM, respectively. Among the two compounds, compound **9**, which consist of an N-OH bond anti to the indole moiety, showed higher inhibitory activity than compound **8**, where the N-OH bond is syn to the indole moiety. 

Among the tested oxime compounds, compound **9** showed the highest potency (the amount of a compound that is needed to develop a defined level of effectiveness) and the efficacy (the maximum inhibitory activity achievable from an applied concentration of each compound) (Table 1). 

### 2.3. In Silico Studies of Indole-3-Carbaldehyde Oxime Derivatives

Molecular docking studies were performed to predict the possible binding modes and interactions between the crystal structure of the urease enzyme of *Helicobacter pylori* (protein) and indole-3-carbaldehyde oxime derivatives (ligands) using the induced-fit docking (FID) method. Mainly, ChemPLP scoring function (Piecewise Linear Potential) in GOLD suite v5.3 was utilized to obtain the fitness score of ligands into the binding site. The resulting ChemPLP score values of oxime derivatives are shown in Table 2. 

The active site of the urease enzyme of *Helicobacter pylori* (Figure S17) contains two Ni^2+^ with an interatomic distance of about 3.5 Å [37]. The coordination sphere of each of the two Ni^2+^ ions is completed by a bridging carbamylated lysine (KCX 219) and a hydroxide [38]. One of the nickel ions is coordinated by two histidine residues (His 248, His 274) and a water molecule and therefore exists as a pentacoordinate metal atom. The other nickel atom consists of two histidine residues (His 136, His 138), a water molecule, and a terminally bound aspartate (Asp 362) [38,39]. 

**Figure 2:**
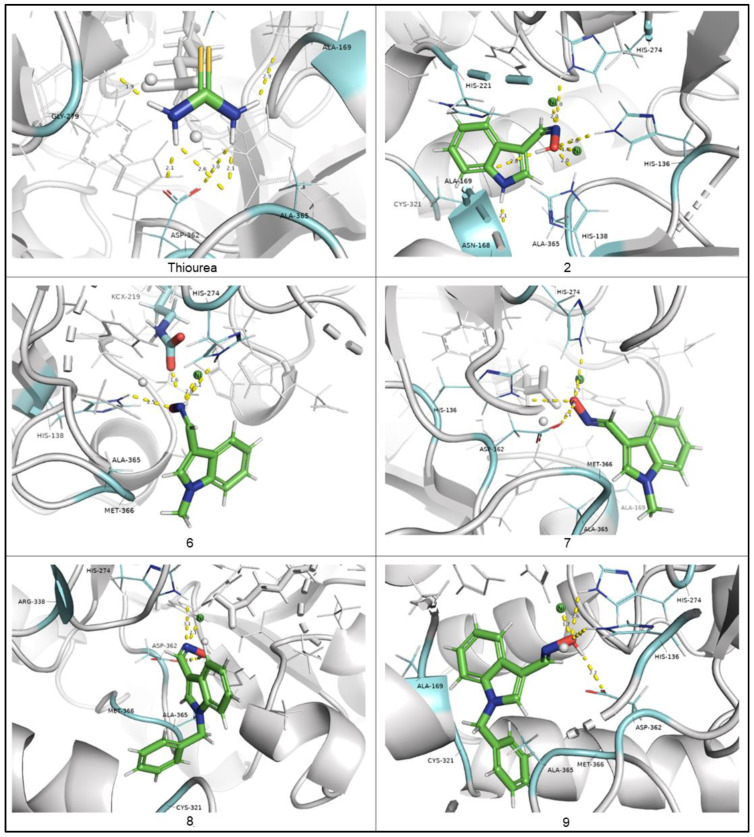
Protein–ligand interaction of the oxime compounds in the active site of the urease enzyme via PyMOL 3D visualizer.

The resulting protein-ligand interactions based on the best binding pose of indole-3-carbaldehyde oxime derivatives (compounds **2**, **6**, **7**, **8**, and **9**) compared to thiourea, visualized by PyMOL and BIOVIA Discovery Studio Visualizer v19, are shown in Figure 2 and Figure S18, respectively. It can be seen that all the structures form hydrogen bonding and pi bond interactions with amino acid residues and metal-acceptor interactions with the two nickel ions in the active site of the urease enzyme. Oxygen and/or nitrogen of oxime group in all the oxime compounds were found to have strong metal–acceptor interactions with Ni 3001 and/or Ni B:3002 atoms within 1.61–2.07 Å and hydrogen bonding with His 274 within 2.62–3.09 Å. The compounds **2**, **6**, and **8**, which have syn configuration, have formed metal–acceptor interactions between Ni 3001 and oxime nitrogen with longer distances, whereas compounds **7** and **9** showed the same interactions with oxygen atoms with less than 1.75 Å. When considering the hydrogen bonding with His 274, the same pattern was observed due to the geometrical differences between syn and anti configurations. The structural differences of the oxime compounds **2**, **6**, **7**, **8**, and **9** (Figure S18) have resulted in different binding interactions with the active site of the urease enzyme and, therefore, different ChemPLP scores.

### 2.4. Drug-Likeness of Indole-3-Carbaldehyde Oxime Derivatives

Drug-likeness is a complex balance of various molecular and pharmacological properties which determines the acceptability of synthetic compounds as drug molecules. Usually, Lipinski’s rule of five and parameters such as logS and TPSA are used to predict the molecular transport properties such as gastrointestinal absorption or blood –brain barrier penetration [40]. According to the Lipinski’s rule of five, a drug-like compound should have a molecular weight of less than 500 Dalton, less than 5 hydrogen bond donors, less than 10 hydrogen bond acceptors and a suitable partition coefficient value, which is less than 5 [41]. The topological polar surface area (TPSA) evaluates the polarity of a molecule by calculating the sum of the contributions of the heteroatoms, including nitrogen, oxygen, and the hydrogen atoms bonded to them [42]. Usually, a drug-like compound possesses a TPSA value at 0~140 Å [42]. The predicted solubility of a compound in water, logS, is the logarithmic value of the molar solubility (mol/L), which should be within the range of −4 to 0.5 [43].

All the oxime compounds (**2**, **6**, **7**, **8**, and **9**) and thiourea were screened to predict their properties such as polarity, lipophilicity, water solubility, and GI absorption using the SwissADME online server (Table 3). According to the results, the TPSA value of compound **2** is higher than that of the other compounds due to the presence of a hydrogen atom in the pyrrole nitrogen. The low logP and high logS values of compound **2** indicate that it is more water soluble and hydrophilic than the other oxime compounds. Since both methyl and benzyl groups are considered as non-polar groups, there is no difference in TPSA values between methylated oximes (compounds **6** and **7**) and benzylated oximes (compounds **8** and **9**). When comparing the logP values of compounds **6**, **7**, **8**, and **9** compounds, higher values resulted for compounds **8** and **9** than compounds **6** and **7** as the former have a non-polar phenyl group. Even though there are variations in the obtained data, all the synthesized oxime compounds obeyed Lipinski’s rule of five and the values of logS and TPSA within the required limits for a drug candidate. 

## 3. Materials and Methods

### 3.1. General 

^1^H and ^13^C nuclear magnetic resonance (NMR) spectra were obtained on BRUKER BioSpin GMBH NMR spectrometer in deuterated acetone unless otherwise noted. Data are reported as follows: chemical shift (ppm); multiplicity (s = singlet, d = doublet, t = triplet, m = multiplet, br = broad); coupling constant (Hz). Anti-urease assay was carried out using Thermos scientific MUITISKAN Sky plate reader and a digital and temperature controllable shaker [(Model Maxturdy-30)- Daihan Scientific, Wonju-Shi, Korea]. A Buchi-4000 Rotary Evaporator was used to remove solvents under reduced pressure. Infrared spectra were recorded in MB 3000 Fourier Transform Infrared Spectrophotometer. Analytical thin-layer chromatography was performed on Merck 60 F_254_ silica gel plates, and isomeric percentages were calculated using ImageJ software (version 1.53e). Column chromatography separations were carried out on silica gel with 200–400 mesh. Chemicals were purchased from Sigma-Aldrich (St. Louis, MO, USA), Sisco Research Laboratories Pvt. Ltd. (Mumbai, India) and other commercial suppliers.

### 3.2. Synthesis of (Z)-N-Hydroxy-1-(1H-indole-3-yl)methanimine (***2***)

#### 3.2.1. Methods a and b 

To a stirred solution of 1*H*-indole-3-carboxaldehyde (**1**) (0.200 g, 1.38 mmol) in 95% EtOH (4.50 mL) in a round bottom flask, NH_2_OH.HCl (0.478 g, 6.89 mmol, 5 eq) and a solution of NaOH [(0.110 g, 2.76 mmol, 2 eq—method **a**), (0.275 g, 6.89 mmol, 5 eq—method **b**)] in distilled water (0.32 mL) was added at 0 °C. The reaction mixture was stirred for 2 h at room temperature. After completion of the reaction, both mixtures were left to stir at room temperature to study the isomerization process (11 days for method **a** and 17 days for method **b**). Each reaction mixture was monitored using TLC every day until the minor isomer (compound **3**) disappeared. Then, the reaction mixture of method **a** was neutralized using a solution of 0.17 mol dm^−3^ NaOH. Both mixtures were diluted with distilled water (9 mL) and extracted with ethyl acetate (EtOAc) (2 × 12 mL) separately. The ethyl acetate layer was separated and dried over anhydrous Na_2_SO_4_. The solvent was evaporated to obtain a peach color residue, and the resulted residue was subjected to silica gel column chromatography (hexane/ethyl acetate 2:1). 

Method **a**—Recrystallized solvent: acetone–water. Yield: 0.196 g (89.07%). White colored crystals. R*_f_* 0.29 (CH_2_Cl_2_/EtOH 10:1). Melting point 179–181 °C. IR (cm^−1^) NH 3350–3400, OH 2700–3150, C=N 1697, N-O 926. ^1^H NMR (400 MHz, Acetone) δ ppm 10.74 (br s, 1H) 10.33 (s, 1H) 8.38 (d, *J* = 2.69 Hz, 1H) 7.87 (d, *J* = 7.70 Hz, 1H) 7.76 (s, 1H) 7.48 (d, *J* = 7.58 Hz, 1H) 7.19 (m, 2H).

Method **b**—Recrystallized solvent: acetone–water. Yield: 0.201 g (91.29%). White colored crystals. R*_f_* 0.30 (CH_2_Cl_2_/EtOH 10:1). Melting point 178–181 °C. IR (cm^−1^) NH 3350–3400, OH 3175–3200, C=N 1625–1650, N-O 900–950.

#### 3.2.2. Methods c and d

To a stirred solution of 1H-indole-3-carboxaldehyde (**1**) (0.100 g, 0.69 mmol) in THF (3 mL), NH_2_OH.HCl (0.239 g, 3.45 mmol, 5 eq) and NaOH ((0.055 g, 1.38 mmol, 2 eq—method **c**), (0.137 g, 3.45 mmol, 5 eq—method **d**)) in distilled water (0.50 mL) were added at 0 °C. The reaction mixtures were stirred for 4 h and 15 min at room temperature. After completion of each reaction, THF was evaporated and diluted with distilled water (4.5 mL). The resultant compounds in the reaction mixtures were extracted to EtOAc (2 × 6.5 mL) separately. Each EtOAc layer was dried over anhydrous Na_2_SO_4,_ and the peach color residues obtained after evaporation of EtOAc were subjected to column chromatographic separation over silica gel (hexane/ethyl acetate 2:1). 

Method **c**—Recrystallized solvent: acetone–water. Yield: 0.076 g (68.93%). White colored crystals. R*_f_* 0.32 (CH_2_Cl_2_/EtOH 10:1). Melting point 179–181 °C. IR (cm^−1^) NH 3350–3385, OH 3150–3200, C=N 1650–1675, N-O 900–950.

Method **d**—Recrystallized solvent: acetone–water. Yield: 0.082 g (74.21%). White colored crystals. R*_f_* 0.30 (CH_2_Cl_2_/EtOH 10:1). Melting point 177–180 °C.

### 3.3. Synthesis of (E)-N-Hydroxy-1-(1H-indole-3-yl)methanimine (***3***)

Since the stability of compound **3** was higher in neutral medium, compound **3** was synthesized and separated out using method **b** under Section 3.2.

Recrystallized solvent: acetone–water. White colored crystals. R*_f_* 0.49 (CH_2_Cl_2_/EtOH 10:1). Melting point 182–185 °C. IR (cm^−1^) NH 3050–3150, OH 2670–3375, C=N 1629, N–O 925. ^1^H NMR (400 MHz, Acetone) δ ppm 10.53 (br s, 1H) 9.68 (s, 1H) 8.34 (s, 1H) 8.12 (d, *J* = 7.92 Hz, 1H) 7.61 (s, 1H) 7.46 (d, *J* = 8.08 Hz, 1H) 7.16 (m, 2H). ^13^C NMR (100 MHz, Acetone) δ ppm 144.73, 137.35, 127.73, 124.85, 122.52, 122.05, 120.13, 111.47, 110.58.

### 3.4. Synthesis of 1-Methyl-1H-indole-3-carbaldehyde (***4***)

To a mixture of 1*H*-indole-3-carbaldehyde (**1**) 0.500 g (3.44 mmol), anhydrous K_2_CO_3_ 0.952 g (6.88 mmol, 2 eq), CH_3_CN (11.5 mL), and DMF (1 mL), methyl iodide 0.52 mL (8.35 mmol, 2.43 eq) was added. The resultant mixture was heated to reflux at 82–84 °C for 16 h. Then, the mixture was cooled to room temperature and filtered. The solution was dried over anhydrous Na_2_SO_4_, and solvents were evaporated under reduced pressure. The crude was recrystallized using ethanol. Yield: 0.478 g (87.28%). Reddish orange powder. *R_f_* 0.91 (CH_2_Cl_2_/EtOH 30:1). Melting point 68–72 °C. IR (cm^−1^) N–CH_3_ 2800–2820, C=O 1600–1650.

### 3.5. Synthesis of (Z/E)-N-Hydroxy-1-(1-methyl-1H-indol-3-yl)methanimine (***6*** and ***7***)

To a stirred solution of 1-methyl-1*H*-indole-3-carbaldehyde (**4**) (0.450 g, 2.83 mmol) in 95% EtOH (8.5 mL) in a 50 mL round bottom flask, 0.982 g (14.13 mmol, 5 eq) of NH_2_OH.HCl and a solution of sodium hydroxide 0.565 g (14.13 mmol, 5 eq) in 1.5 mL of distilled water were added at 0–5 °C. The reaction mixture was stirred for four hours at room temperature. After completion of the reaction, the solvent was evaporated under reduced pressure. Then, the reaction mixture was diluted with distilled water (20 mL) and extracted with EtOAc (2 × 27 mL), dried over anhydrous Na_2_SO_4_, and evaporated the solvent to afford aldoxime as a mixture of *Z* and *E* isomers. Orange-brown solid, *R_f_* 0.34 (**6**), 0.66 (**7**) (DCM/EtOH 10:1). The resultant mixture was subjected to column chromatography on silica gel using a mixture of hexane and ethyl acetate (2:1)

(**6**) Recrystallization solvent: acetone–water. Yield: 0.219 g (54.77%). White colored crystals. R*_f_* 0.52 (CH_2_Cl_2_/EtOH 10:1). Melting point 112–115 °C. IR (cm^−1^) OH 2750–3200, CH 2862, C=N 1635, N-O 941. ^1^H NMR (400 MHz, Acetone) δ ppm 10.32 (s, 1H) 8.26 (s, 1H) 7.85 (d, *J* = 7.82 Hz, 1H) 7.73 (s, 1H) 7.46 (d, *J* = 8.07 Hz, 1H) 7.25 (t, *J* = 7.58 Hz, 1H) 7.17 (m, 1H) 3.91 (s, 3H).

(**7**) Recrystallization solvent: acetone–water. Yield: 0.168 g (42.04%). White colored crystals. R*_f_* 0.80 (CH_2_Cl_2_/EtOH 10:1). Melting point 126–129 °C. IR (cm^−1^) OH 2800–3325, CH 2908, C=N 1636, N-O 941. ^1^H NMR (400 MHz, Acetone) δ ppm 9.64 (s, 1H) 8.28 (s, 1H) 8.09 (d, *J* = 8.07 Hz, 1H) 7.50 (s, 1H) 7.42 (d, *J* = 8.31 Hz, 1H) 7.25 (t, *J* = 1.71 Hz, 1H) 7.13 (m, 1H) 3.86 (s, 3H).

### 3.6. Synthesis of 1-Benzyl-1H-indole-3-carbaldehyde (***5***)

A mixture of 1*H*-indole-3-carbaldehyde (**1**) 0.300 g (2.067 mmol), benzyl chloride 0.40 mL (3.51 mmol, 1.7 eq) and anhydrous K_2_CO_3_ 0.571 g (4.13 mmol, 2 eq) in CH_3_CN (8 mL) and DMF (0.1 mL, 1.34 mmol, 0.65 eq) were heated to reflux at 82–84 °C for 12 h. After the reaction, the resultant mixture was cooled to room temperature and filtered. The solution was dried over anhydrous Na_2_SO_4,_ and solvents were evaporated under reduced pressure. The obtained residue was recrystallized using a mixture of ethanol –water to afford compound **5**. Yield: 0.383 g (78.81%). Pale orange yellowish powder. *R_f_* 0.81 (DCM/EtOH 30:1). Melting point 121–122 °C. IR (cm^−1^) C=O 1635–1650.

### 3.7. Synthesis of (Z/E)-1-(1-Benzyl-1H-indol-3-yl)-N-hydroxymethanimine (***8*** and ***9***)

To a stirred solution of 1-benzyl-1*H*-indole-3-carbaldehyde (**5**) (0.090 g, 0.38 mmol) in 95% ethanol (7 mL), NH_2_OH.HCl (0.133 g, 1.91 mmol, 5 eq) and a saturated solution of NaOH (0.076 g, 1.91 mmol, 5 eq) in 0.5 mL of distilled water was added at 0–5 °C. The reaction mixture was stirred for 2 h at room temperature. After the evaporation of ethanol and subsequent dilution with water (4 mL), the mixture was extracted with ethyl acetate two times (2 × 6 mL). The combined organic layers were dried over anhydrous Na_2_SO_4_, and the residue obtained after evaporation of the solvent was subjected to column chromatographic separation over silica gel (hexane/EtOAc 2:1) to obtain two isomers, (**8**) and (**9**). Grayish yellow powder. *R_f_* 0.62 (**8**), 0.88 (**9**) (CH_2_Cl_2_/EtOH 10:1).

(**8**) Recrystallization solvent: DMF–water. Yield: 0.034 g (40.12%). White colored flakes. *R_f_* 0.57 (CH_2_Cl_2_/EtOH 10:1). Melting point 132–134 °C. IR (cm^−1^) OH 2675–3250, C-H 2924, C=N 1627, N-O 933. ^1^H NMR (400 MHz, Acetone) δ ppm 10.39 (s, 1H) 8.44 (s, 1H) 7.88 (t, *J* = 1.96Hz, 1H) 7.77 (s, 1H) 7.47 (t, *J* = 1.22 Hz, 1H) 7.34–7.28 (m, 2H) 7.23–7.16 (m, 5H) 5.55 (s, 2H). ^13^C NMR (100 MHz, Acetone) δ ppm 138.17, 137.75, 135.46, 134.10, 128.64, 127.54, 127.06, 122.21, 120.43, 116.33, 110.39, 106.43, 49.83.

(**9**) Recrystallization solvent: DMF–water. Yield: 0.047 g (56.20%). White colored flakes. *R_f_* 0.83 (CH_2_Cl_2_/EtOH 10:1). Melting point 140–143 °C. IR (cm^−1^) OH 2850–3350, C-H 2908, C=N 1643, N-O 948. ^1^H NMR (400 MHz, Acetone) δ ppm 9.70 (s, 1H) 8.31 (s, 1H) 8.11 (d, *J* = 7.82 Hz, 1H) 7.65 (s, 1H) 7.42 (d, *J* = 8.31 Hz, 1H) 7.25–7.36 (m, 5H) 7.20–7.13 (m, 2H) 5.47 (s, 2H). ^13^C NMR (100 MHz, Acetone) δ ppm 144.35, 137.79, 137.35, 131.08, 128.63, 127.53, 127.04, 125.61, 122.63, 122.36, 120.38, 110.19, 49.66.

### 3.8. Urease Inhibitory Assay

Modified Berthelot method [35,36] was used for the determination of the amount of produced ammonia, and thereby for the quantification of the urease inhibitory activities of the synthesized oxime derivatives (compounds **2**, **6**, **7**, **8**, and **9**) by measuring their absorbance at 630 nm. All the test compounds and thiourea were prepared by dissolving 2 mg of each compound in 20 mL of 1.25% DMF - buffer solution (pH = 7.2, Na_2_HPO_4_ = 0.067 mol dm^−3^, KH_2_PO_4_ = 0.033 mol dm^−3^). DMF doesn’t show any inhibitory effect on urease activity [44]. Initially, a 96 well plate filled with 10 μL of urease enzyme (0.01 U) and 10–70 μL of the test compound was placed in the plate reader and incubated at 37 °C for 15 min. (Concentration of each sample was changed by varying the volume of test compounds from 10 to 70 μL.) Then, to each well, 10 μL of the buffer, 10 μL of EDTA (0.025 mol dm^−3^), and 10 μL of urea (0.64 mol dm^−3^) were added. After that, 80–20 μL of distilled water was added accordingly to adjust the total volume to 130 μL and incubated in plate reader for another 20 min at 37 °C. Then 35 μL of each coloring agents ((coloring agent A—1% (*w*/*v*) phenol, 0.005% (*w*/*v*) sodium nitroprusside and coloring agent B—0.5% (*w*/*v*) NaOH, 0.05% (*v*/*v*) NaOCl)) were introduced to each well, and the plate was incubated in plate reader for another 50 min at 37 °C for the development of indophenol blue color. At the end of the 50 min, the absorbance of each sample was measured at the absorbance wavelength of 630 nm. A control test was conducted to quantify the total hydrolysis of urea in the presence of urease (C). Negative control was carried out to quantify the spontaneous hydrolysis of urea in the absence of the urease enzyme (N). For each sample (T_1–7_), a negative control test (N_1–7_) was performed in the absence of the urease enzyme. All experiments were performed thrice. The percentage inhibition of the urease enzyme was calculated using Equation (1).

(1) $$percentage\;inhibition=\left[\frac{\left[A_{C}-A_{N}\right]-\left[A_{T_{1}}-A_{N_{1}}\right]}{\left[A_{C}-A_{N}\right]}\right]\times 100$$

where

$A_{C}$ = Absorbance of the control sample (*C*)
$A_{N}$ = Absorbance of the negative control sample (*N*)
$A_{T_{1-7}}$ = Absorbance of the test sample (*T*_1_ to *T*_7_)
$A_{N_{1-7}}$*=* Absorbance of the negative control of the test sample (*N*_1_ to *N*_7_)

### 3.9. Molecular Docking Studies

The crystal structure of *H*. *pylori* urease (protein) in complex with acetohydroxamic acid (IE9Y) was obtained in a PDB format from the RCSB Protein Data Bank [45]. The complexed ligand (acetohydroxamic acid) and water molecules were removed, and the protein was protonated by adding hydrogen atoms using the GOLD suite v5.3 program [46,47]. The active site of the protein was identified using the same software. The synthesized molecules (ligands) and thiourea were prepared using ChemDraw Professional and CS Chem3D Ultra softwares (version 20.0.0.41, PerkinElmer Infoematics, Waltham, MA, USA, 2020) [48]. Energies of all the prepared molecules were minimized using Avogadro 1.2.0 [49] by choosing MMFF94s as the force field and conjugate gradient option. Using the same software, all PDB files were converted to MDL Mol format. Molecular docking was performed using the GOLD suite v5.3 program by selecting all atoms within a range of 10 Å, and Chemscore_kinase was used as the configuration template. Gold fitness values were obtained for the best ranking pose by analyzing 30 poses of each ligand using the ChemPLP score function. Binding interactions of ligands with the active site of the urease enzyme were studied using BIOVIA Discovery Studio Visualizer (version 19.1.0.18287, BIOVIA, Dassault Systèmes, San Diego, CA, USA, 2018)) and PyMOL. (The PyMOL Molecular Graphics System, Version 2.5.2 Schrödinger, LLC, New York, NY, USA, 2021).

### 3.10. Drug-Likeness Prediction of Indole-3-Carbaldehyde Oxime Derivatives

Drug-likeness of the oxime compounds (**2**, **6**, **7**, **8**, and **9**) and thiourea was evaluated by calculating physicochemical properties, lipophilicity, water solubility, pharmacokinetics, drug-likeness, and medicinal chemistry parameters using SwissADME online server http://www.swissadme.ch/ (accessed on 24 August 2021). Initially, 2D structures of the indole-3-carbaldehyde oxime molecules and thiourea were prepared using ChemDraw Professional [48] and converted to Structural Data File format. Then each SDF file was uploaded to the SwissADME online web server and predicted the above-mentioned parameters. 

## 4. Conclusions

Even though various therapeutics are available to treat gastrointestinal diseases, there is still no effective drug available for the management of gastritis caused by *Helicobacter pylori*. Inhibition of *Helicobacter pylori* urease which is a commonly used approach to treat gastrointestinal infections, was the basis in this study. A series of syn and anti isomers of *N*-substituted indole-3-carbaldehyde oxime derivatives (compounds **2**, **6**, **7**, **8**, and **9**) was synthesized by changing the substituents attached to the indole nitrogen. For compounds **2** and **3**, isomerization was studied using time-resolved TLC. In the case of unsubstituted derivatives (**2** and **3**), only syn isomer (**2**) was stable, whereas *N*-methyl and *N*-benzyl derivatives (compounds **6**–**9**) were obtained as syn and anti isomers. All the tested compounds were identified using melting points determination, FT-IR, and ^1^H- NMR techniques. Additionally, ^13^C NMR analysis was performed for compounds **8** and **9**. To evaluate the urease enzyme inhibitory activity, compounds **2**, **6**, **7**, **8**, and **9** were subjected to in vitro anti-urease assay. All the tested oxime derivatives have shown better anti-urease activity compared to thiourea. Among them, compounds **8** and **9** showed higher inhibitory activity. According to the in silico studies, compounds **2**, **6**, **7**, **8**, and **9** have shown their ability to bind with the active site and inhibit the urease enzyme. To achieve better inhibitory activities, syn and anti isomers of *N*-substituted indole-3-carbaldehyde oxime derivatives can be used with further structural optimization. Therefore, to develop a more potent anti-urease drug, indole oxime can be used as a lead compound. Further SAR studies can lead to identifying novel urease inhibitors for the treatment of *Helicobacter pylori* infections.

## Figures and Tables

**Table 1:** IC_50_ values and the maximum percentage of inhibitory values of thiourea and the tested oxime compounds **2**, **6**, **7**, **8**, and **9**.

Compound	IC_50_ Value (mM)	Maximum Percentage of Inhibition
**Thiourea**	0.2387 ± 0.0048	85.14
**2**	0.1412 ± 0.0075	67.81
**6**	0.1405 ± 0.0045	77.05
**7**	0.1530 ± 0.0016	57.17
**8**	0.0516 ± 0.0035	83.78
**9**	0.0345 ± 0.0008	86.09

**Table 2:** Molecular docking results of thiourea and the oxime compounds **2**, **6**, **7**, **8**, and **9**.

Compound	ChemPLP Score
**Thiourea**	23.27
**2**	60.50
**6**	62.06
**7**	64.92
**8**	80.47
**9**	82.17

**Table 3:** Predicted drug-likeness properties of thiourea and the oxime compounds **2**, **6**, **7**, **8**, and **9**.

Compound	MW ^a^/(g/mol)	TPSA ^b^ (Å)	Consensus Log P_o/w_ ^c^	Log S ^d^ (mol/L)	n-HA ^e^	n-HD ^f^	n-Violations ^g^	GI Absorption ^h^
**Thiourea**	76.12	84.13	−0.43	0.29	2	4	0	high
**2**	160.17	48.38	1.72	−2.49	3	2	0	high
**6**	174.20	37.52	1.71	−2.78	3	1	0	high
**7**	174.20	37.52	1.74	−2.78	3	1	0	high
**8**	250.30	37.52	2.99	−4.65	3	1	0	high
**9**	250.30	37.52	3.01	−4.65	3	1	0	high

^a^ Molecular weight; ^b^ topological polar surface area; ^c^ log of the octanol/water partition coefficient; ^d^ log of the aqueous solubility; ^e^ number of hydrogen bond acceptors; ^f^ number of hydrogen bond donors; ^g^ number of violations of Lipinski’s rule of five; ^h^ gastrointestinal absorption.

## Data Availability

The data presented in this study are available in supplementary material.

## Supplementary Materials

The following are available online, Figure S1: Time resolved TLC analysis of *N*-hydroxy-1-(1*H*-indole-3-yl)methanimine (compounds **2** and **3**), Figure S2: FT-IR spectrum of (*Z*)-*N*-hydroxy-1-(1*H*-indole-3-yl)methanimine (Compound **2**), Figure S3: FT-IR spectrum of (*E*)-*N*-hydroxy-1-(1*H*-indole-3-yl)methanimine (Compound **3**), Figure S4: FT-IR spectrum of (*Z*)-*N*-hydroxy-1-(1-methyl-1*H*-indol-3-yl)methanimine (Compound **6**), Figure S5: FT-IR spectrum of (*E*)-*N*-hydroxy-1-(1-methyl-1*H*-indol-3-yl)methanimine (Compound **7**), Figure S6: FT-IR spectrum of (*Z*)-1-(1-benzyl-1*H*-indol-3-yl)-*N*-hydroxymethanimine (Compound **8**), Figure S7: FT-IR spectrum of (*E*)-1-(1-benzyl-1*H*-indol-3-yl)-*N*-hydroxymethanimine (Compound **9**), Figure S8: ^1^H-NMR spectrum of (*Z*)-*N*-hydroxy-1-(1*H*-indole-3-yl)methanimine (Compound **2**), Figure S9: ^1^H-NMR spectrum of (*E*)-*N*-hydroxy-1-(1*H*-indole-3-yl)methanimine (Compound **3**), Figure S10: ^1^H-NMR spectrum of (*Z*)-*N*-hydroxy-1-(1-methyl-1*H*-indol-3-yl)methanimine (Compound **6**), Figure S11: ^1^H-NMR spectrum of (*E*)-*N*-hydroxy-1-(1-methyl-1*H*-indol-3-yl)methanimine (Compound **7**), Figure S12: ^1^H-NMR spectrum of (*Z*)-1-(1-benzyl-1*H*-indol-3-yl)-*N*-hydroxymethanimine (Compound **8**), Figure S13: ^1^H-NMR spectrum of (*E*)-1-(1-benzyl-1*H*-indol-3-yl)-*N*-hydroxymethanimine (Compound **9**), Figure S14: ^13^C-NMR spectrum of (*E*)-*N*-hydroxy-1-(1*H*-indole-3-yl)methanimine (Compound **3**), Figure S15: ^13^C-NMR spectrum of (*Z*)-1-(1-benzyl-1*H*-indol-3-yl)-*N*-hydroxymethanimine (Compound **8**), Figure S16: ^13^C-NMR spectrum of (*E*)-1-(1-benzyl-1*H*-indol-3-yl)-*N*-hydroxymethanimine (Compound **9**), Figure S17: Active site of the urease enzyme of *Helicobacter pylori*, Figure S18: Protein-ligand interaction of the oxime compounds in the active site of the urease enzyme via PyMOL 3D visualizer, Scheme S1: Oximation and the isomerization of aldoximes, Table S1: Chemical shifts comparison of oxime compounds, Table S2: Percentage inhibition of indole-3-carbaldehyde oxime derivatives.

## Sample Availability

Samples of the compound are available from the authors.
