# Supplementary material for: Synthesis, In Silico Studies, and Evaluation of Syn and Anti Isomers of N-Substituted Indole-3-carbaldehyde Oxime Derivatives as Urease Inhibitors against Helicobacter pylori
Source: Molecules. 2021 Nov 3;26(21):6658. doi: 10.3390/molecules26216658 (PMC8588131; doi:10.3390/molecules26216658)
Supplement: Supplementary file 1 [file molecules-26-06658-s001.zip › molecules-1420560-supplementary.pdf]

molecules

Supplementary information

Synthesis, *in silico* studies and evaluation of *syn* and *anti* isomers of *N*-substituted indole-3-carbaldehyde oxime derivatives as urease inhibitors against *Helicobacter pylori*

Ishani P. Kalatuwawege [1], Medha J. Gunaratna [2,*] and Dinusha N. Udukala [1]

[1] College of Chemical Sciences, Institute of Chemistry Ceylon, Rajagiriya 10100, Sri Lanka
[2] Department of Chemistry, University of Kelaniya, Kelaniya 10300, Sri Lanka
* Correspondence: medhagunaratna@kln.ac.lk

Content

Figure S1 Time resolved TLC analysis of *N*-hydroxy-1-(1*H*-indole-3-yl)methanimine (compounds **2** and **3**)
Scheme S1 Oximation and the isomerization of aldoximes
Table S1 Chemical shifts comparison of oxime compounds
Figure S2 FT-IR spectrum of (*Z*)-*N*-hydroxy-1-(1*H*-indole-3-yl)methanimine (Compound **2**)
Figure S3 FT-IR spectrum of (*E*)-*N*-hydroxy-1-(1*H*-indole-3-yl)methanimine (Compound **3**).
Figure S4 FT-IR spectrum of (*Z*)-*N*-hydroxy-1-(1-methyl-1*H*-indol-3-yl)methanimine (Compound **6**)
Figure S5 FT-IR spectrum of (*E*)-*N*-hydroxy-1-(1-methyl-1*H*-indol-3-yl)methanimine (Compound **7**)
Figure S6 FT-IR spectrum of (*Z*)-1-(1-benzyl-1*H*-indol-3-yl)-*N*-hydroxymethanimine (Compound **8**)
Figure S7 FT-IR spectrum of (*E*)-1-(1-benzyl-1*H*-indol-3-yl)-*N*-hydroxymethanimine (Compound **9**)
Figure S8 ^{1}H-NMR spectrum of (*Z*)-*N*-hydroxy-1-(1*H*-indole-3-yl)methanimine (Compound **2**)
Figure S9 ^{1}H-NMR spectrum of (*E*)-*N*-hydroxy-1-(1*H*-indole-3-yl)methanimine (Compound **3**)
Figure S10 ^{1}H-NMR spectrum of (*Z*)-*N*-hydroxy-1-(1-methyl-1*H*-indol-3-yl)methanimine (Compound **6**)
Figure S11 ^{1}H-NMR spectrum of (*E*)-*N*-hydroxy-1-(1-methyl-1*H*-indol-3-yl)methanimine (Compound **7**)
Figure S12 ^{1}H-NMR spectrum of (*Z*)-1-(1-benzyl-1*H*-indol-3-yl)-*N*-hydroxymethanimine (Compound **8**)
Figure S13 ^{1}H-NMR spectrum of (*E*)-1-(1-benzyl-1*H*-indol-3-yl)-*N*-hydroxymethanimine (Compound **9**)
Figure S14 ^{13}C-NMR spectrum of (*E*)-*N*-hydroxy-1-(1*H*-indole-3-yl)methanimine (Compound **3**)
Figure S15 ^{13}C-NMR spectrum of (*Z*)-1-(1-benzyl-1*H*-indol-3-yl)-*N*-hydroxymethanimine (Compound **8**)
Figure S16 ^{13}C-NMR spectrum of (*E*)-1-(1-benzyl-1*H*-indol-3-yl)-*N*-hydroxymethanimine (Compound **9**)
Table S2 Percentage inhibition of indole-3-carbaldehyde oxime derivatives
Figure S17 Active site of the urease enzyme of *Helicobacter pylori*
Figure S18 Protein-ligand interaction of the oxime compounds in the active site of the urease enzyme via PyMOL 3D visualizer.

1. Isomerization of *N*-hydroxy-1-(1*H*-indole-3-yl)methanimine (compounds 2 and 3)

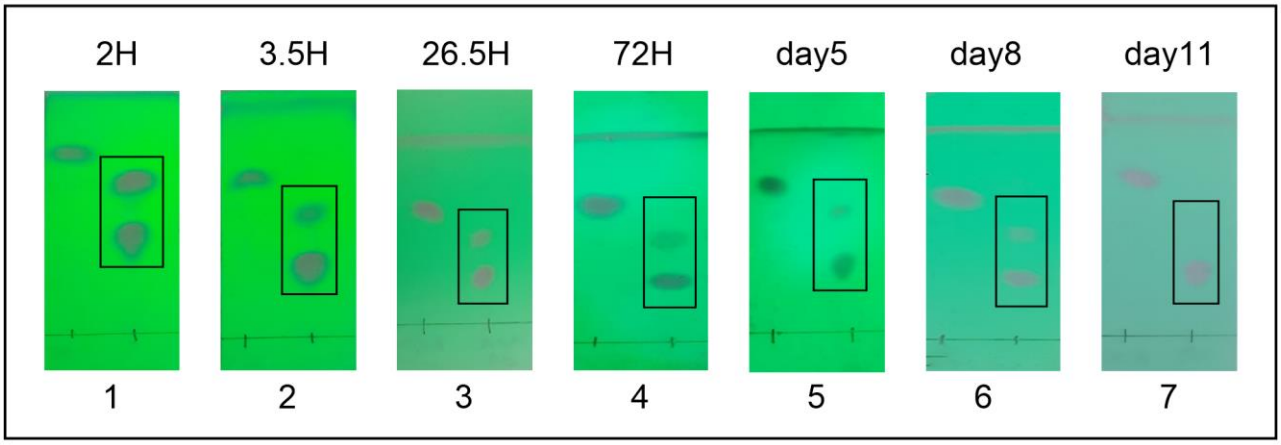

Figure S1. Time resolved TLC analysis of *N*-hydroxy-1-(1*H*-indole-3-yl)methanimine (compounds **2** and **3** as *syn* and *anti* isomers respectively). In this figure, bottom spot indicates the compound **2** which have a higher polarity and upper spot indicates the less polar compound **3**.

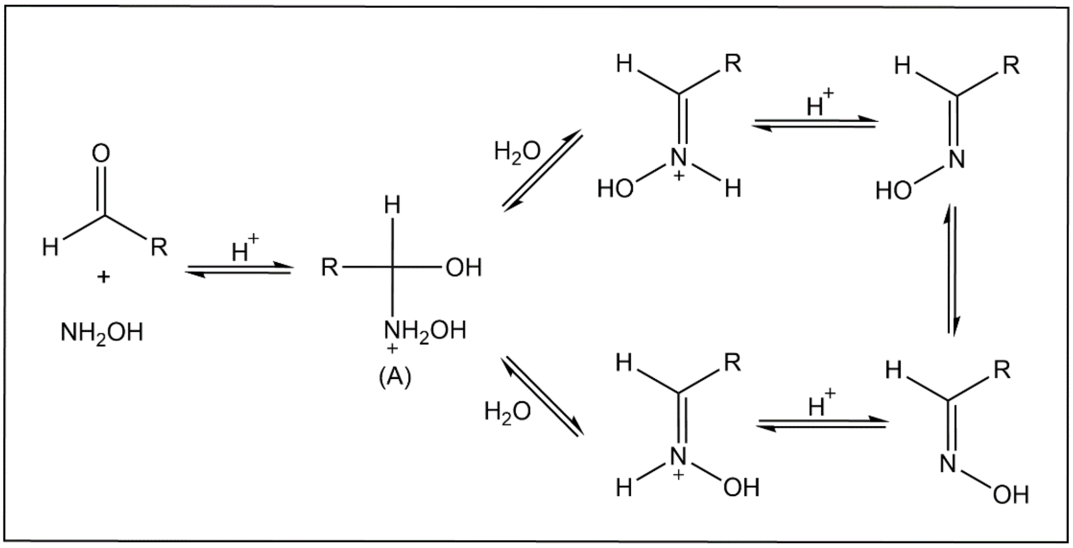

Scheme S1. Oximation and the isomerization of aldoximes.

According to the literature, there are two steps in the reaction mechanism of the synthesis of oximes. In the first step, an in situ deprotonation of hydroxylammonium ion by NaOH occurs. In the second step, nucleophilic attack of the hydroxylamine to 1*H*-indole-3-carbaldehyde, followed by the condensation reaction occurs. For the in-situ deprotonation, a stoichiometric ratio of NH_2OH.HCl and NaOH (1:1) should be used. Since in the reaction **a**, an excess amount of NH_2OH.HCl was used, it can be assumed that this excess amount of NH_2OH.HCl has resulted the reaction mixture to be acidic that leads to isomerization. As shown in the Scheme S1, both hydrolysis and isomerization can be achieved through a tetrahedral carbinolamino intermediate (A).

2. Chemical shifts comparison of indole-3-carbaldehyde oxime derivatives

Table S1. Chemical shifts comparison of oxime compounds.

Compound	Chemical shift			
	**H-2**	**H-4**	**H-8**	**OH**
2 (*syn*)	8.3819	7.8719	7.7674	10.3374
3 (*anti*)	7.6142	8.1241	8.3444	9.6848
6 (*syn*)	8.2614	7.8534	7.7314	10.3264
7 (*anti*)	7.5008	8.0977	8.2881	9.6425
8 (*syn*)	8.4420	7.8826	7.7760	10.3918
9 (*anti*)	7.6592	8.1101	8.3133	9.7055

In compound **6**, chemical shift of the OH proton (10.32 ppm) and H-2 proton (8.26 ppm) is higher than the chemical shift values of the same protons in the compound **7** as 9.64 ppm and 7.50 ppm respectively. The higher chemical shift for the H-2 proton of compound **6** is due to the higher de-shielding effect by the OH group which is in close proximity. Due to the same reason, H-4 and H-8 protons in the compound **7** (8.09 ppm and 8.28 ppm) have shown higher chemical shift values than compound **6** (7.85 ppm and 7.73 ppm). It was noted that among these two protons, the chemical shift of H-8 is higher than the chemical shift of H-4 in the compound **7** whereas in the compound **6** the chemical shift of H-4 is higher. For the compounds **2**,**3**,**8** and **9**, a similar chemical shift pattern was observed.

3. FT-IR spectral data of indole-3-carbaldehyde oxime derivatives

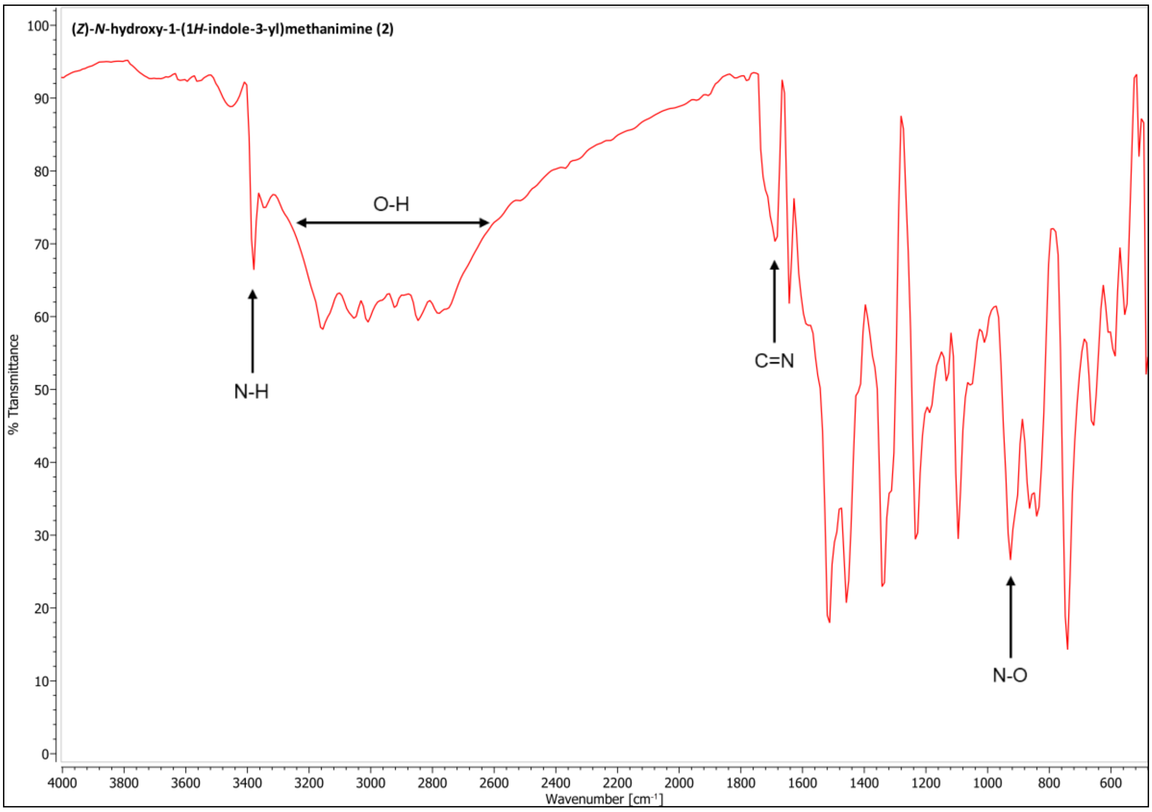

Figure S2. FT-IR spectrum of (*Z*)-*N*-hydroxy-1-(1*H*-indole-3-yl)methanimine (Compound **2**).

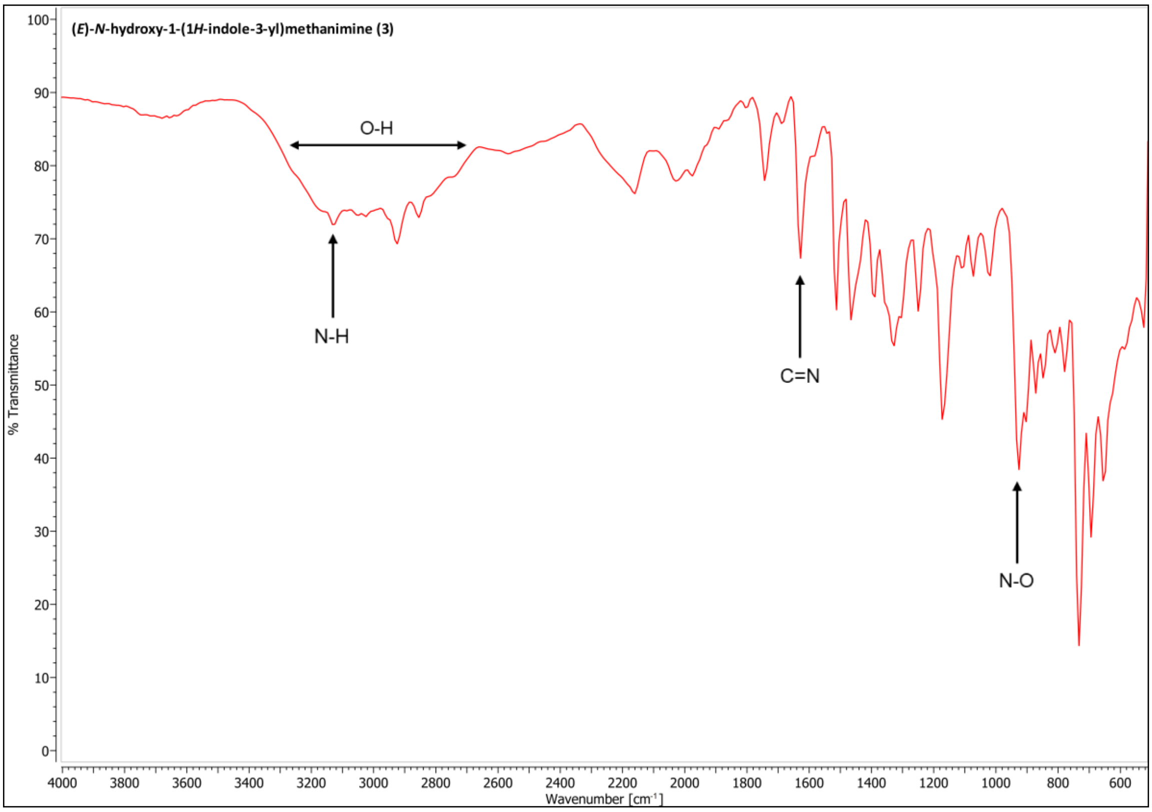

Figure S3. FT-IR spectrum of (*E*)-*N*-hydroxy-1-(1*H*-indole-3-yl)methanimine (Compound **3**).

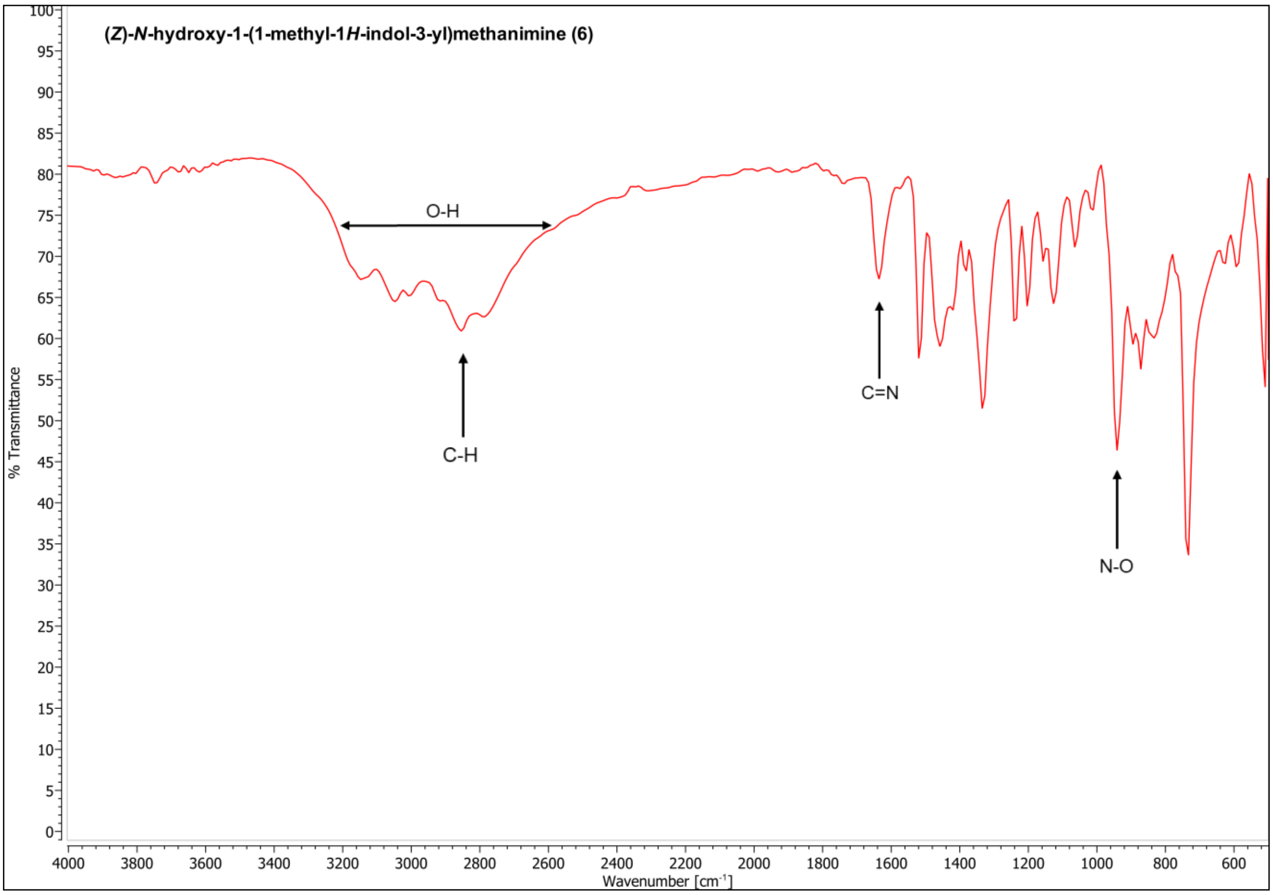

Figure S4. FT-IR spectrum of (*Z*)-*N*-hydroxy-1-(1-methyl-1*H*-indol-3-yl)methanimine (Compound **6**).

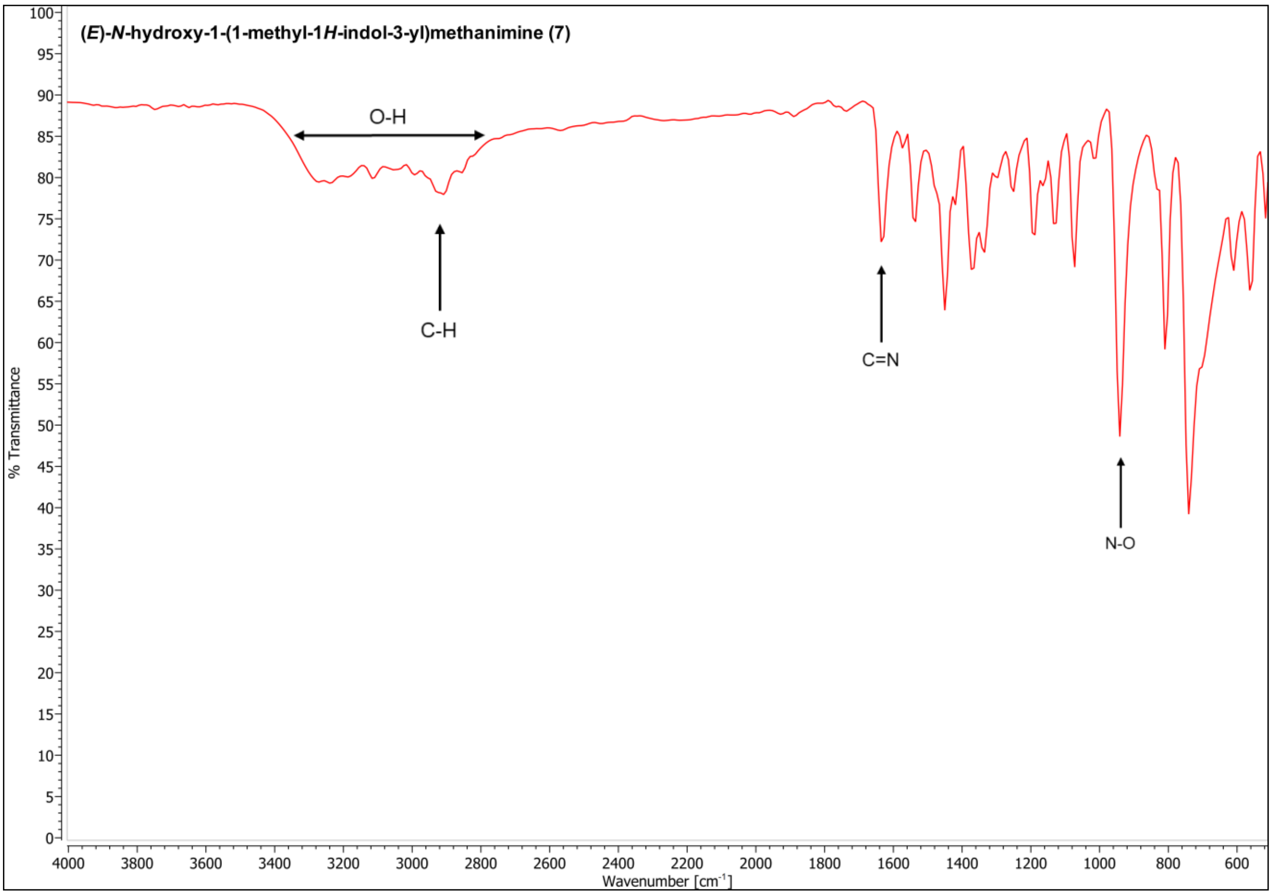

Figure S5. FT-IR spectrum of (E)-N-hydroxy-1-(1-methyl-1H-indol-3-yl)methanimine (Compound **7**).

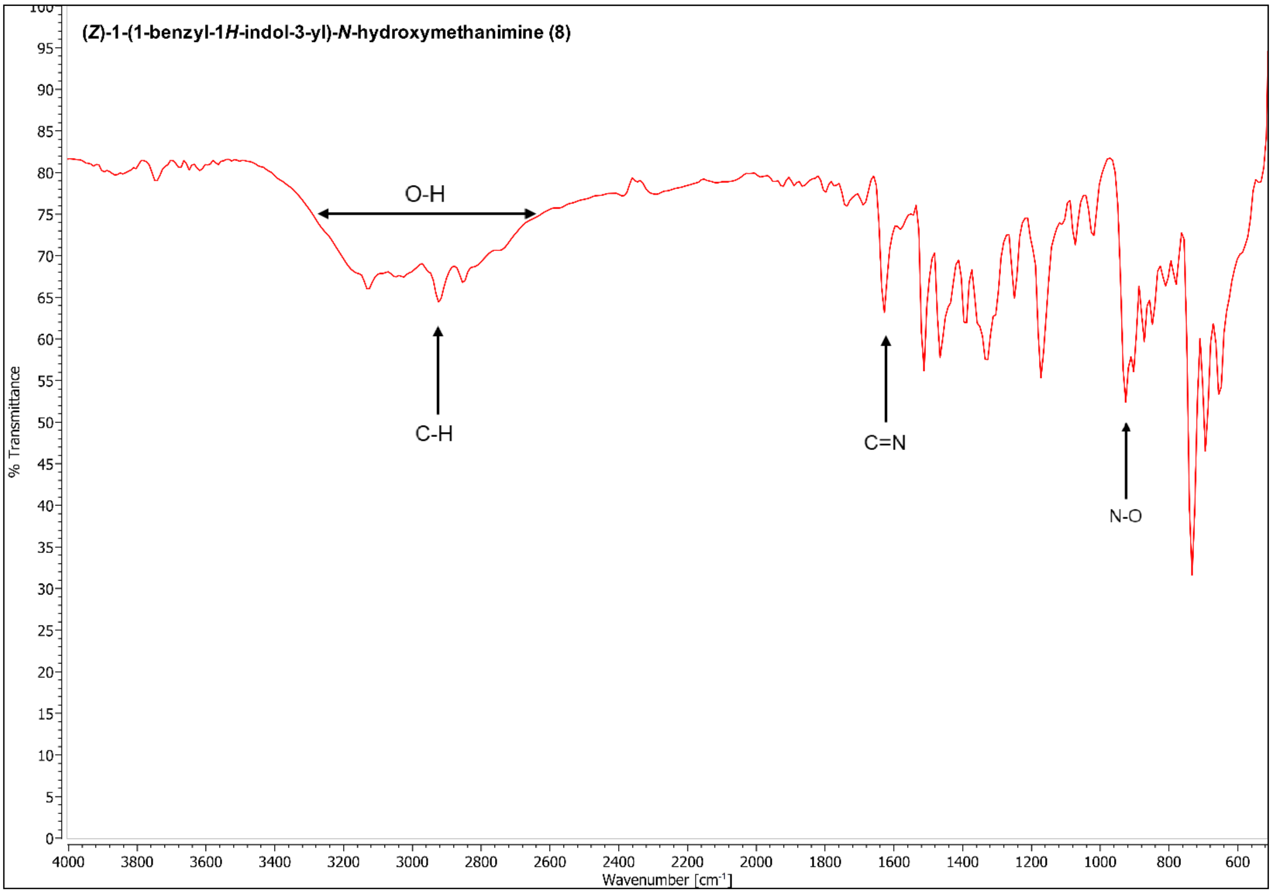

Figure S6. FT-IR spectrum of (*Z*)-1-(1-benzyl-1*H*-indol-3-yl)-*N*-hydroxymethanimine (Compound **8**).

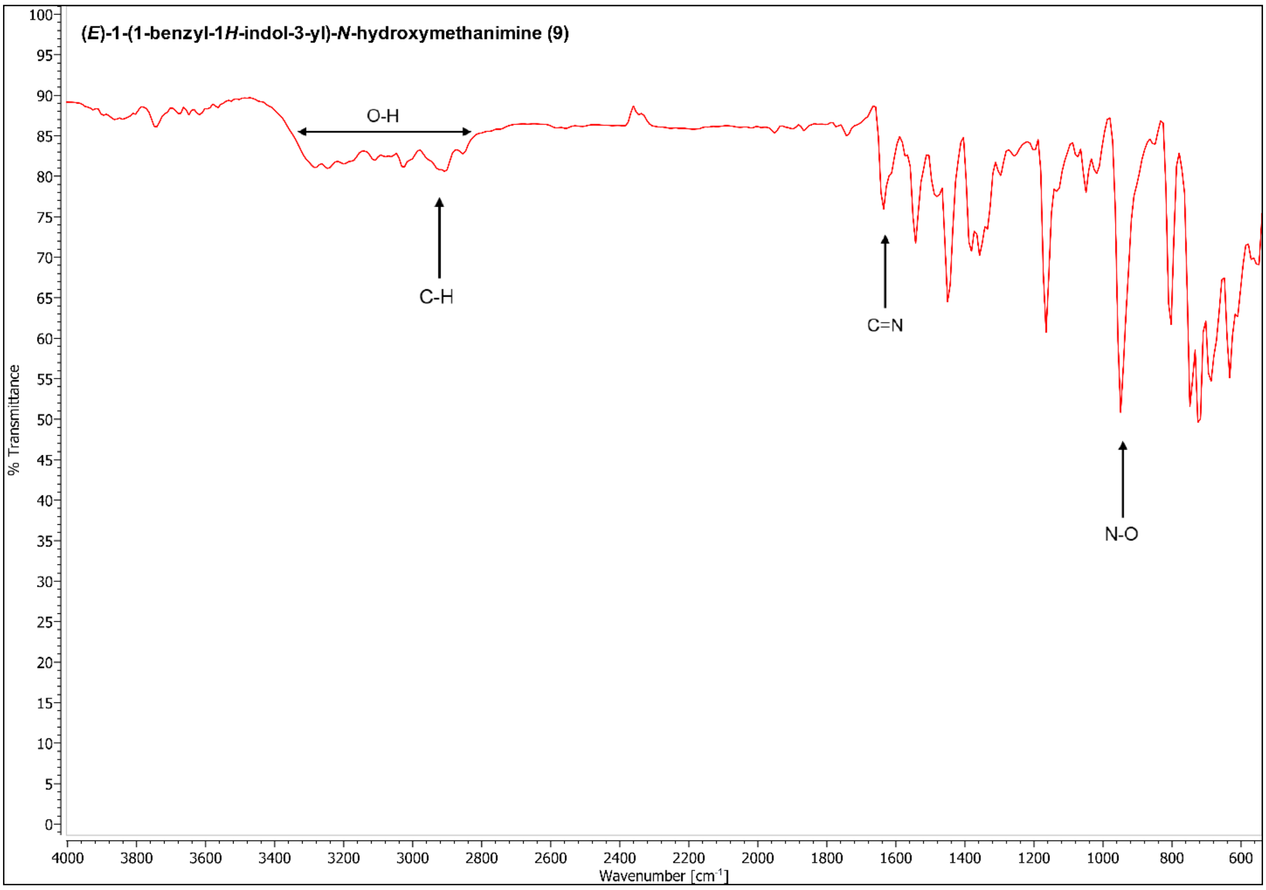

Figure S7. FT-IR spectrum of (*E*)-1-(1-benzyl-1*H*-indol-3-yl)-*N*-hydroxymethanimine (Compound **9**).

4. ^{1}H and ^{13}C NMR spectral data of indole-3-carbaldehyde oxime derivatives

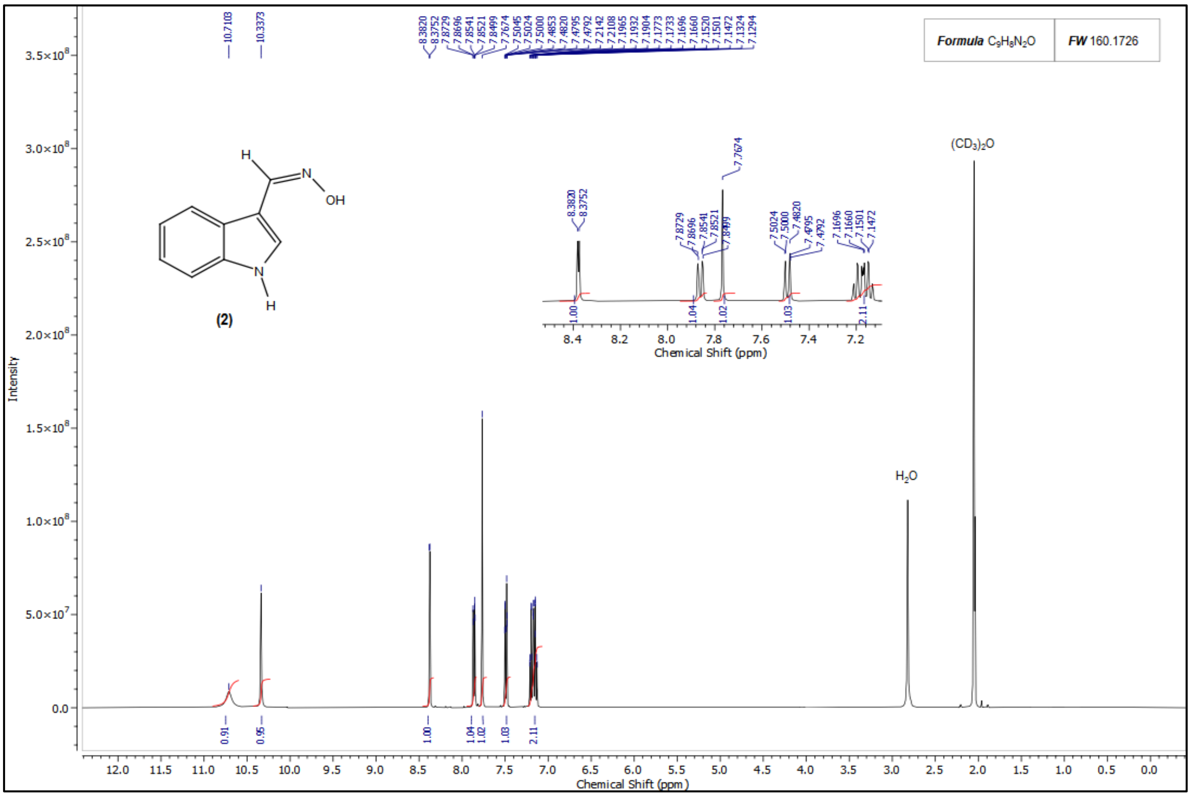

Figure S8. ^{1}H-NMR spectrum of (*Z*)-*N*-hydroxy-1-(1*H*-indole-3-yl)methanimine (Compound **2**).

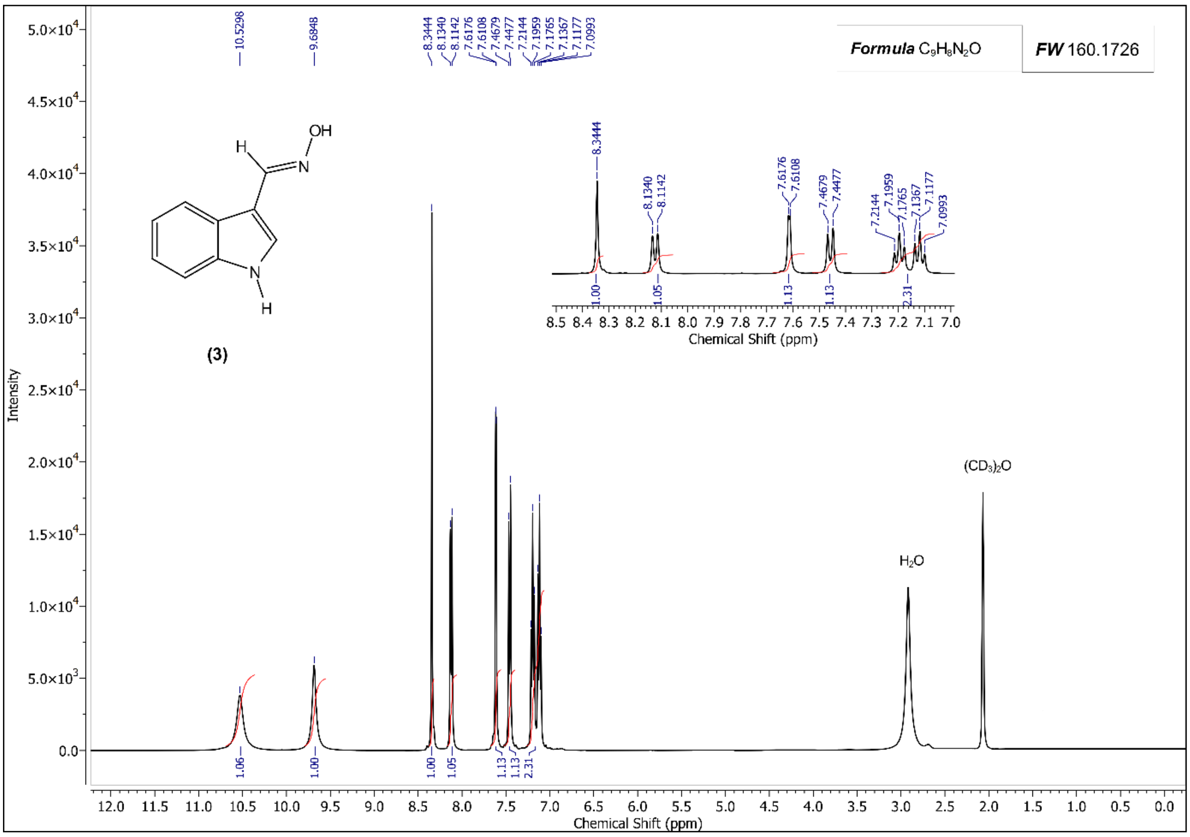

Figure S9. ^{1}H-NMR spectrum of (*E*)-*N*-hydroxy-1-(1*H*-indole-3-yl)methanimine (Compound **3**)

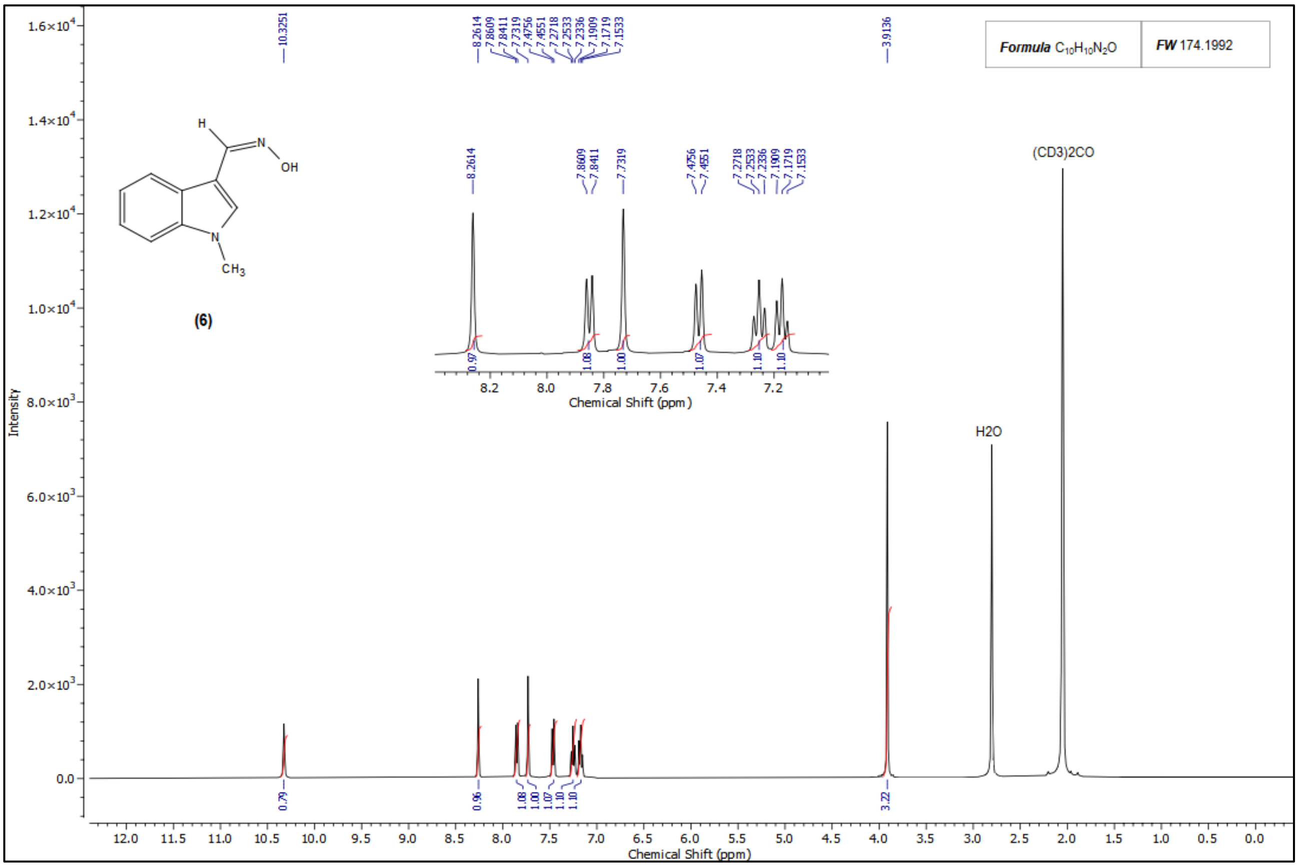

Figure S10. ^{1}H-NMR spectrum of (*Z*)-*N*-hydroxy-1-(1-methyl-1*H*-indol-3-yl)methanimine (Compound **6**).

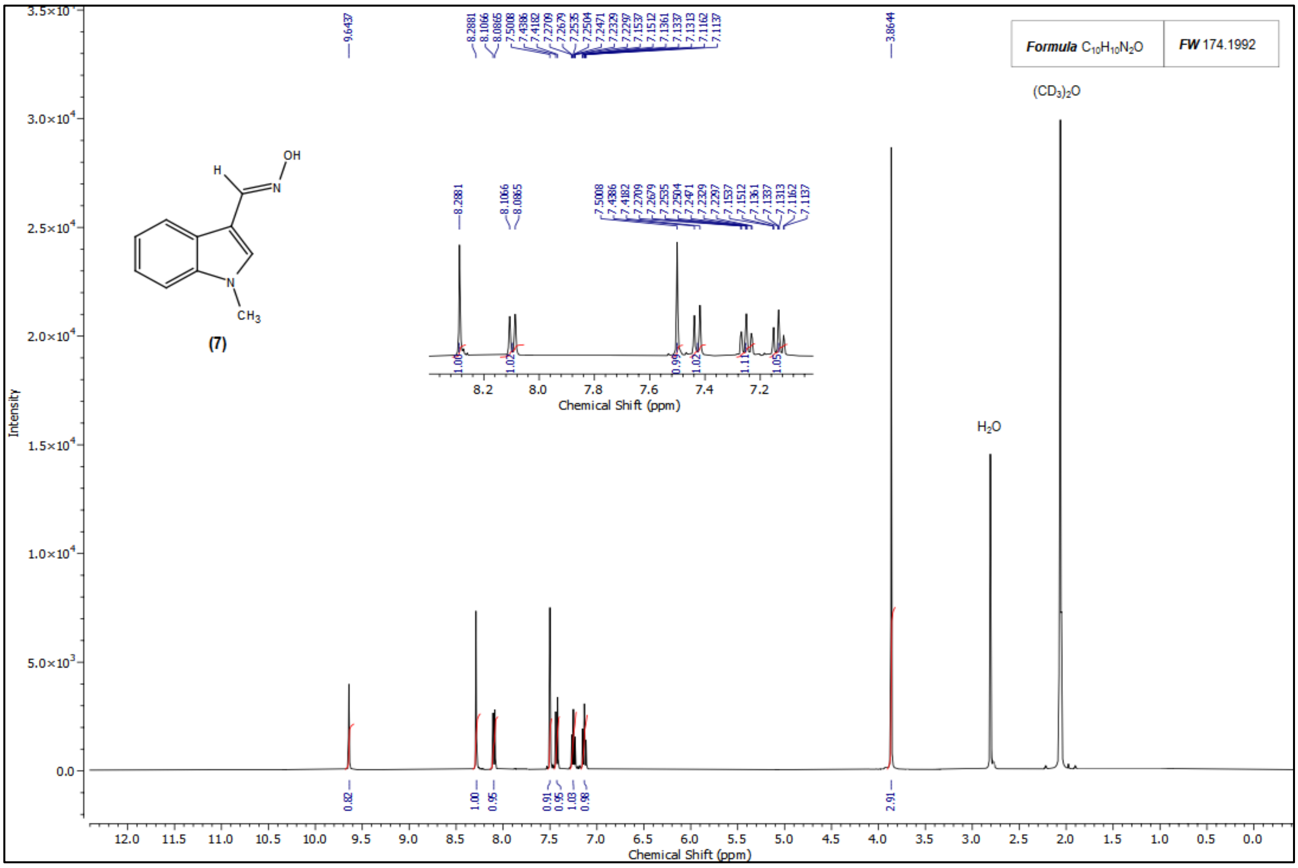

Figure S11. ^{1}H-NMR spectrum of (*E*)-*N*-hydroxy-1-(1-methyl-1*H*-indol-3-yl)methanimine (Compound **7**).

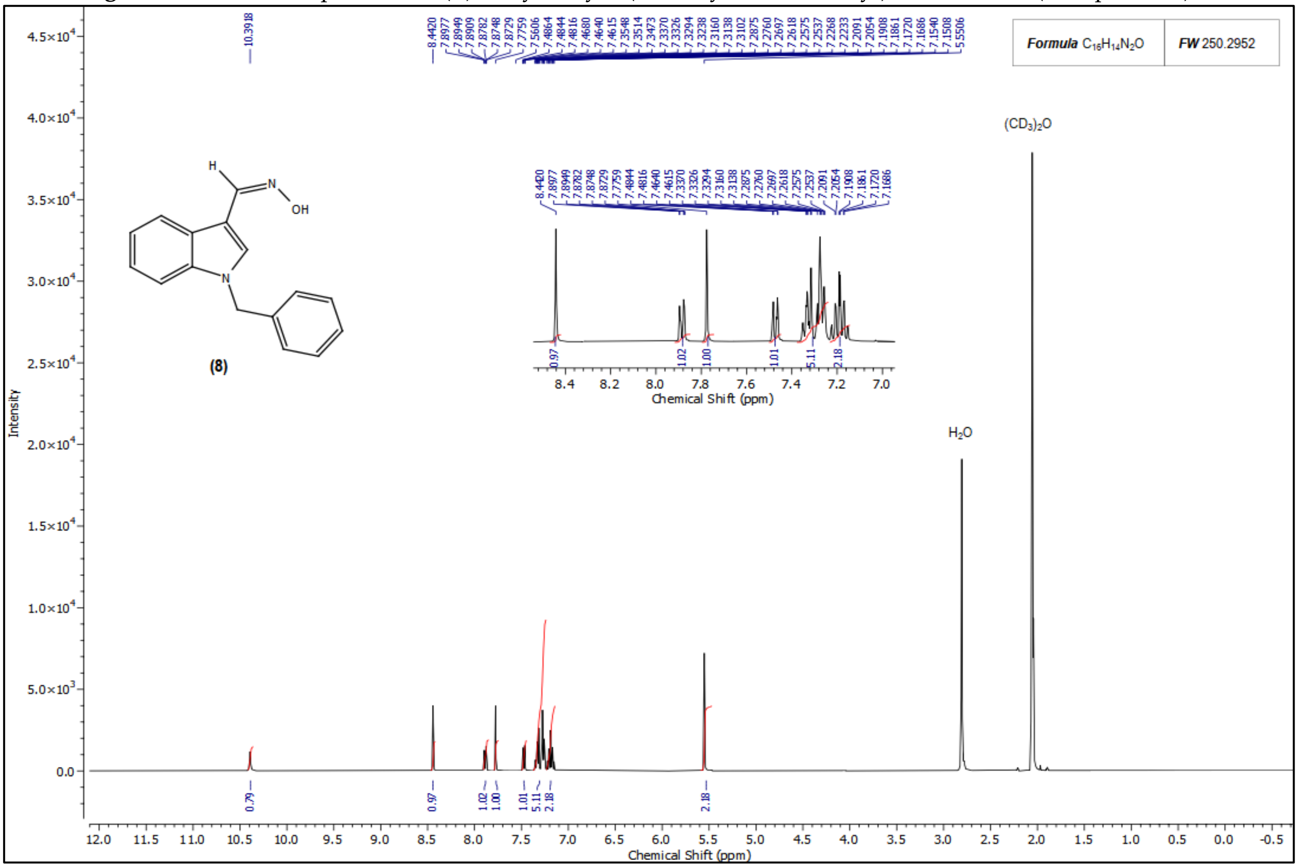

Figure S12. ^{1}H-NMR spectrum of (*Z*)-1-(1-benzyl-1*H*-indol-3-yl)-*N*-hydroxymethanimine (Compound **8**).

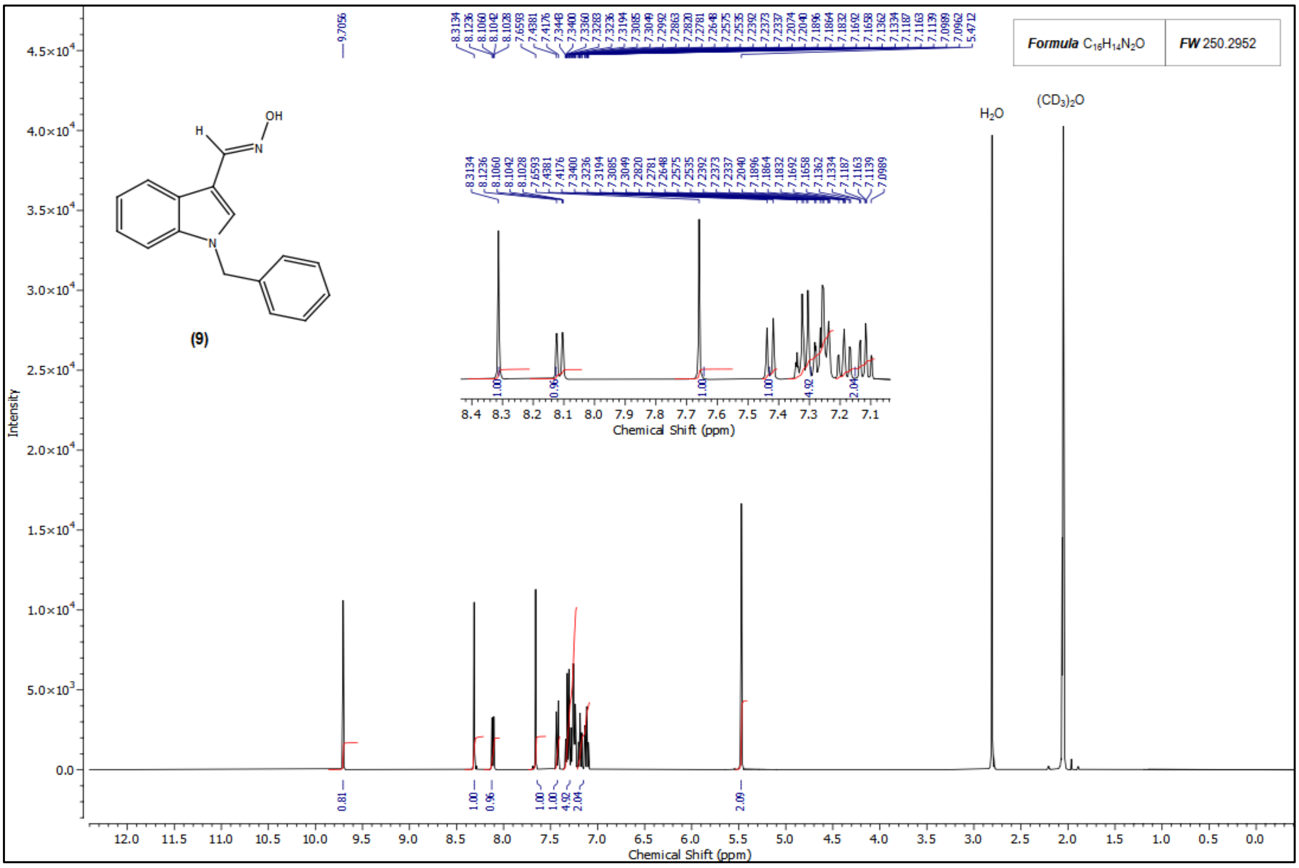

Figure S13. ^{1}H-NMR spectrum of (*E*)-1-(1-benzyl-1*H*-indol-3-yl)-*N*-hydroxymethanimine (Compound **9**).

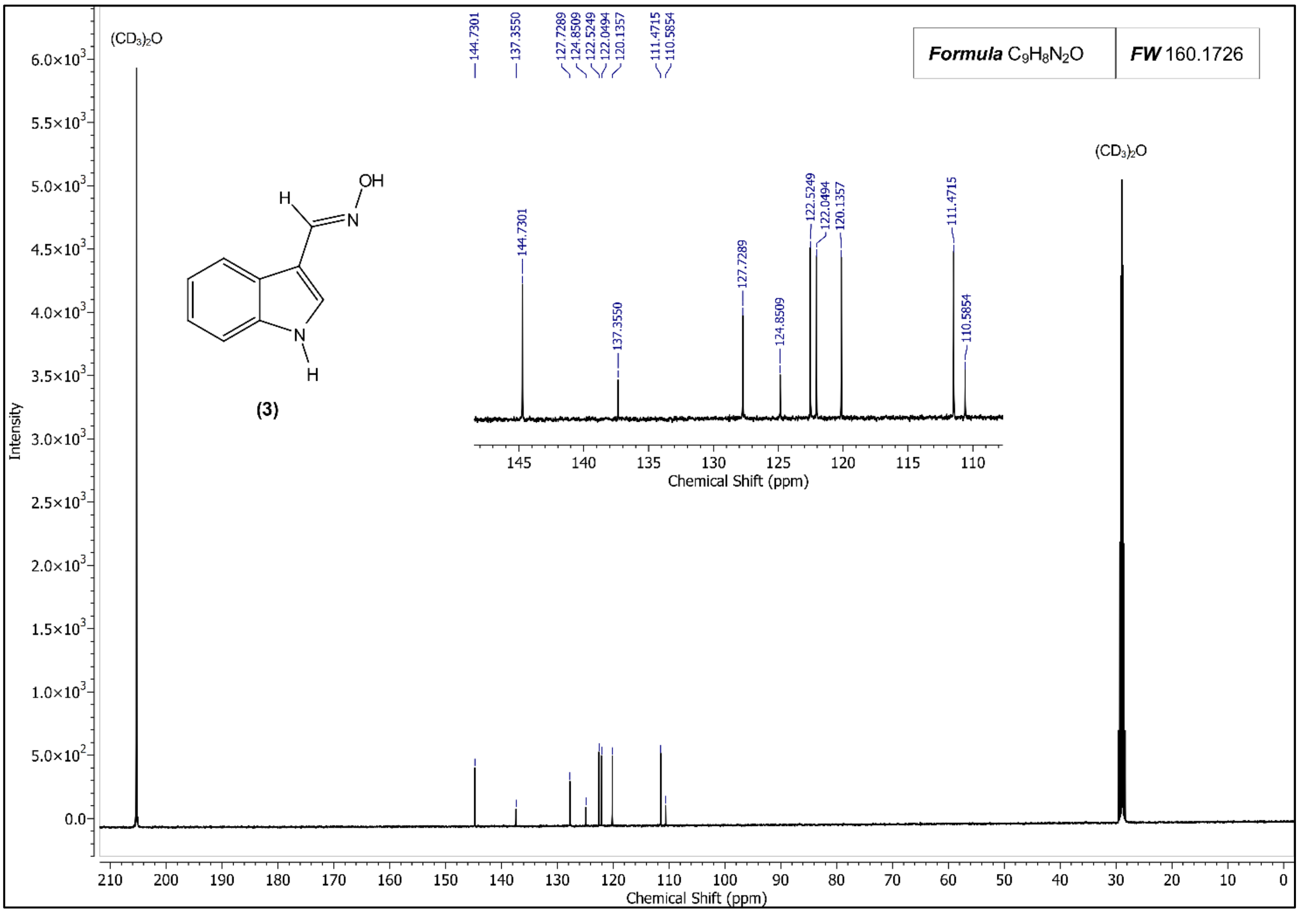

Figure S14. ^{13}C-NMR spectrum of (*E*)-*N*-hydroxy-1-(1*H*-indole-3-yl)methanimine (Compound **3**).

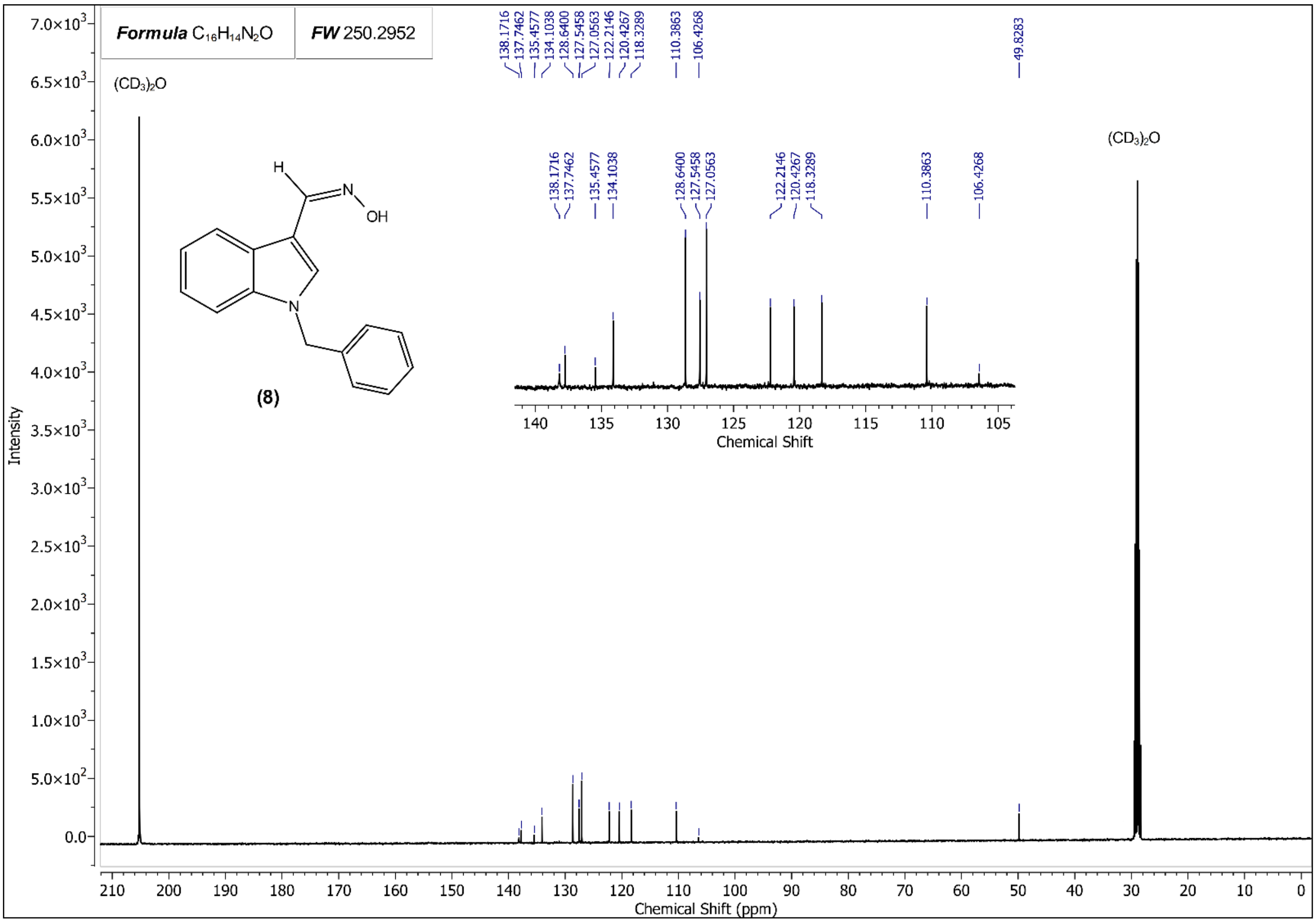

Figure S15. ^{13}C-NMR spectrum of (*Z*)-1-(1-benzyl-1*H*-indol-3-yl)-*N*-hydroxymethanimine (Compound **8**).

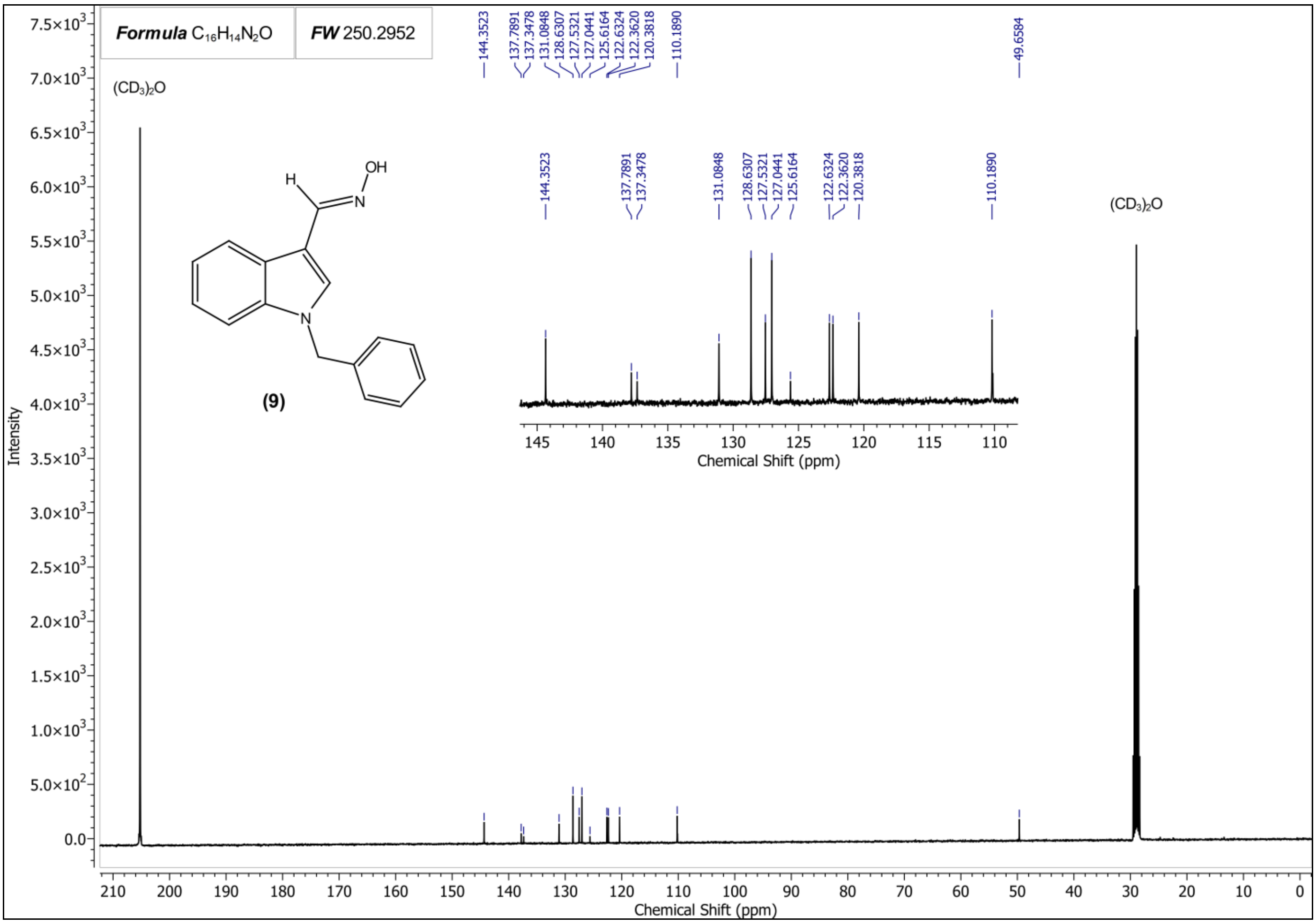

Figure S16. ^{13}C-NMR spectrum of (*E*)-1-(1-benzyl-1*H*-indol-3-yl)-*N*-hydroxymethanimine (Compound **9**)

5. Urease inhibitory assay of indole-3-carbaldehyde oxime derivatives

Table S2. Percentage inhibiton of tested indole-3-carbaldehyde oxime derivatives.

compound	Percentage inhibition (% ± SEM)						
	at 5.00 µg/mL	at 10.00 µg/mL	at 15.00 µg/mL	at 20.00 µg/mL	at 25.00 µg/mL	at 30.00 µg/mL	at 35.00 µg/mL
thiourea	11.24 ± 0.28	22.61 ± 0.49	38.41 ± 0.63	61.54 ± 0.53	74.36 ± 0.50	81.49 ± 0.67	85.14 ± 0.74
2	17.46 ± 0.71	24.56 ± 0.53	29.28 ± 0.52	39.40 ± 0.65	57.27 ± 0.63	64.87 ± 0.93	67.81 ± 0.79
6	24.57 ± 1.22	28.15 ± 0.81	29.91 ± 0.90	40.38 ± 0.24	55.76 ± 0.60	73.07 ± 0.35	77.05 ± 1.41
7	22.74 ± 0.93	31.87 ± 0.59	38.14 ± 1.25	44.38 ± 0.35	48.92 ± 0.67	53.01 ± 0.85	57.17 ± 0.36
8	36.36 ± 0.79	44.39 ± 0.70	58.52 ± 0.58	67.99 ± 1.01	76.49 ± 1.24	79.64 ± 0.88	83.78 ± 0.99
9	33.06 ± 1.23	59.35 ± 0.70	72.38 ± 0.77	77.61 ± 1.55	83.21 ± 0.82	84.89 ± 0.47	86.09 ± 0.72

6. *In silico* studies of indole-3-carbaldehyde oxime derivatives

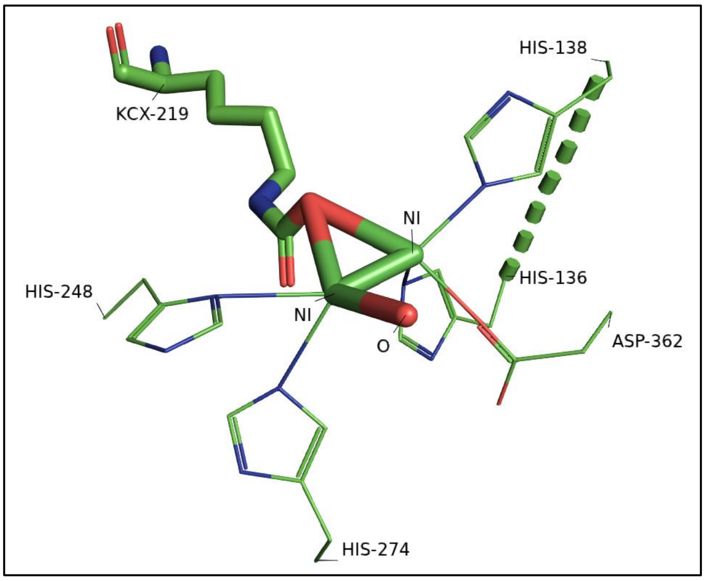

Figure S17. Active site of the ureaese enzyme of *Helicobacter pylori*.

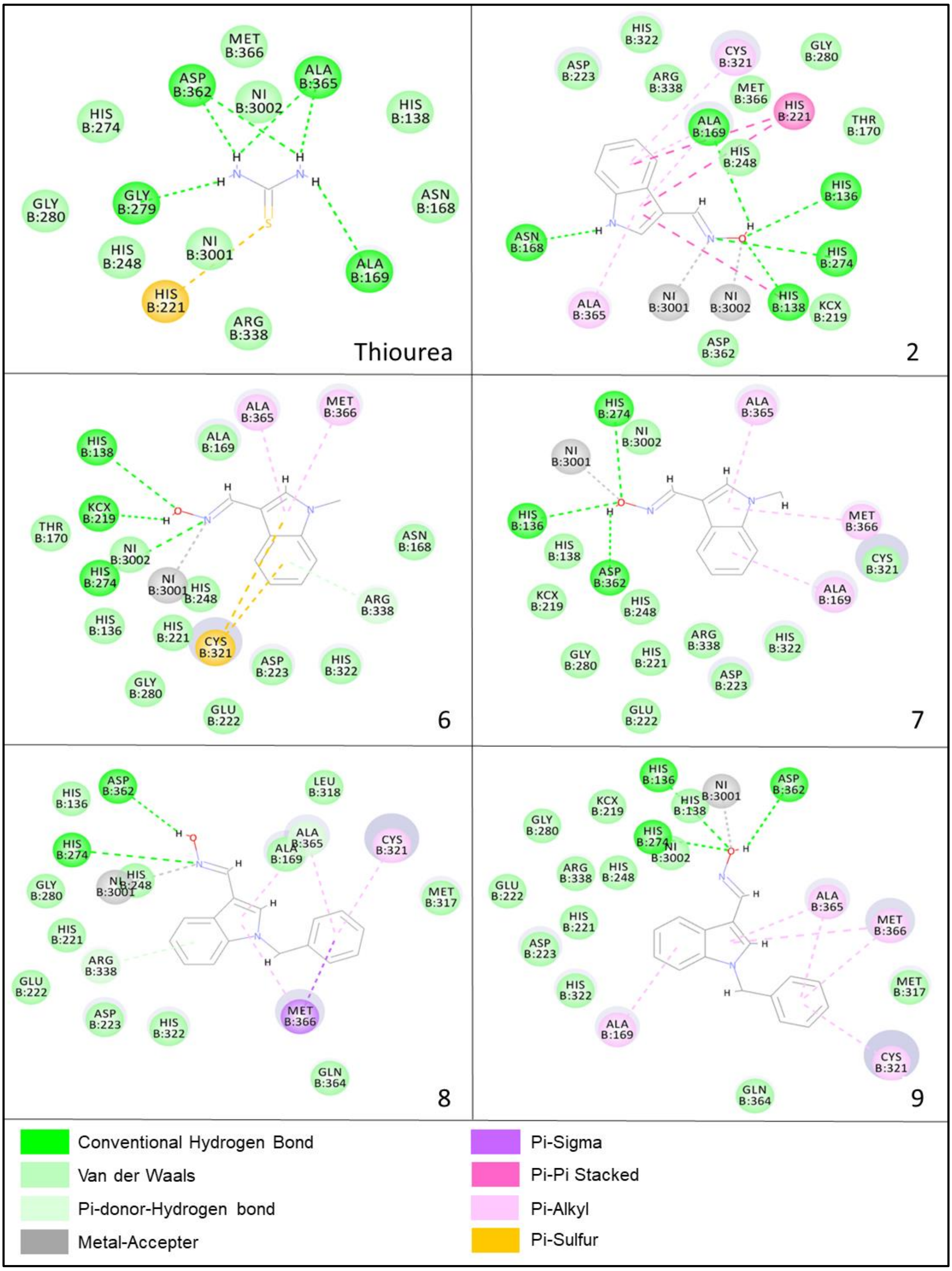

Figure S18. Protein-ligand interactions of the oxime compounds in the active site of the urease enzyme via BIOVIA visual studio.

Hydrogen and oxygen of the oxime group and hydrogen bonded to the pyrrole nitrogen of compound **2** showed hydrogen bonding interactions with backbone oxygen of Ala 169 (2.85 Å), ε_2 hydrogen of His 136 (2.85 Å), and backbone oxygen of Asn 168 (2.14 Å) respectively. Further, the imidazole ring of His 221 formed Pi-Pi stacking with both pyrrole and benzene rings of the compound **2**. The docking orientation of compound **6** showed hydrogen bond interactions between oxime's hydrogen and oxygen in the side chain of KCX 219 within 1.65 Å, and between oxygen and ε_2 nitrogen of His 138 at a distance of 2.62 Å. The compound **7** was also found to possess additional hydrogen bonding between hydrogen of oxime group and δ_1 oxygen in the side chain of Asp 362 within 2.10 Å and between oxygen and ε_2 hydrogen of His 136 within 2.73 Å. Similarly, compounds **8** and **9** possessed, hydrogen bonding between oximes' hydrogen and δ_2/ δ_1 oxygen atoms in the side chain of Asp 362 residue at 2.11 Å and 2.10 Å distances respectively. Other than that, compound **9** showed another hydrogen bonding between oxygen and ε_2 hydrogen of His 274 in a distance of 2.62 Å. The presence of benzyl group attached to the pyrrole nitrogen in compounds **8** and **9** has further strengthen their binding to the active site via Pi-Alkyl/Sigma interactions with Ala 365, Cys 321 and Met 366 within the range of 4.05 – 4.99 Å. According to resulted ChemPLP scores, *anti* compounds **7** and **9** had higher fitness scores than the *syn* compounds **2**, **6**, and **8**.
